# Proportionate clinical burden of respiratory diseases in Indian outdoor services and its relationship with seasonal transitions and risk factors: The results of SWORD survey

**DOI:** 10.1371/journal.pone.0268216

**Published:** 2022-08-18

**Authors:** Bharat Bhushan Sharma, Sheetu Singh, Krishna Kumar Sharma, Arvind Kumar Sharma, K. P. Suraj, Tariq Mahmood, Kumar Utsav Samaria, Surya Kant, Nishtha Singh, Tejraj Singh, Aradhana Singh, Rajeev Gupta, Parvaiz A. Koul, Sundeep Salvi, Virendra Singh, SWORD study group

**Affiliations:** 1 Department of Medicine, Division of Allergy and Pulmonary Medicine, S.M.S. Medical College, Jaipur, Rajasthan, India; 2 Department of Respiratory Medicine, Lung Center, Rajasthan Hospital, Jaipur, Rajasthan, India; 3 Department of Pharmacology, Lal Bahadur College of Pharmacy, Rajasthan University of Health Sciences, Jaipur, Rajasthan, India; 4 Department of Community Medicine, Mahatma Gandhi Medical College, Jaipur, Rajasthan, India; 5 Department of Pulmonary Medicine, Government Medical College, Kozhikode, Kerala, India; 6 Department of Pulmonary Medicine, MLN Medical College, Prayagraj (Allahabad), Uttar Pradesh, India; 7 Pulmonology Division, Agrim Hospital, Varanasi, Uttar Pradesh, India; 8 Department of Respiratory Medicine, King George’s Medical University, Lucknow, Uttar Pradesh, India; 9 Department of Respiratory Medicine, Asthma Bhawan, Jaipur, Rajasthan, India; 10 Department of Research Division, Asthma Bhawan, Jaipur, Rajasthan, India; 11 Department of Medicine, S.M.S. Medical College, Jaipur, Rajasthan, India; 12 Department of Preventive Cardiology and Internal Medicine, Eternal Heart Care Centre and Research Institute, Mount Sinai New York Affiliate, Jaipur, Rajasthan, India; 13 Department of Internal and Pulmonary Medicine, SKIMS, Srinagar, Jammu and Kashmir, India; 14 Pulmocare Research and Education Foundation, Pune, Maharashtra, India; 15 Department of Pulmonary Medicine, Director, Rajasthan Hospital & Asthma Bhawan, Jaipur, Rajasthan, India; AIIMS: All India Institute of Medical Sciences, INDIA

## Abstract

**Background:**

The Global Burden of Disease data suggest that respiratory diseases contribute to high morbidity in India. However, the factors responsible for high morbidity are not quite clear. Therefore, the Seasonal Waves Of Respiratory Disorders (SWORD) study was planned to estimate the point prevalence due to respiratory diseases in Indian OPD services and its association with risk factors and change in seasons.

**Methods:**

In this point prevalence observational multicenter study conducted during 2017–18, participating physicians recorded information of consecutive patients in response to a questionnaire. The study was conducted on four predetermined days representing transition of Indian seasons i.e., February (winter), May (summer), August (monsoon), and November (autumn).

**Results:**

The eligible number of patients from across 302 sites in India was 25,177. The mean age of study population was 46.1±18.1 years, 14102(56.0%) were males and 11075(44.0%) females. The common diagnoses were: asthma(29.8%), chronic obstructive pulmonary disease (COPD),15.6%, respiratory tract infections (RTIs),11.3%, and tuberculosis(8.7%). All these conditions showed significant seasonal trends (Asthma 31.4% autumn vs. 26.5% summer, COPD 21.1% winter vs. 8.1% summer, RTIs 13.3% winter vs. 4.3% summer, and tuberculosis 12.5% autumn vs. 4.1% summer, p<0.001 for each respectively). After adjustment for risk factors, asthma was significantly associated with exposure to molds (OR:1.12,CI:1.03–1.22), pet animals (OR:1.07,CI:1.01–1.14), recent-travel (OR:1.22,CI:1.13–1.32), and rain-wetting (OR:1.27,CI:1.15–1.40); and RTIs with rain-wetting (OR:1.53,CI:1.34–1.74), and recent-travel (OR:1.17,CI:1.05–1.30).

**Conclusions:**

The SWORD study showed wide seasonal variations in outpatient attendance of patients with common respiratory conditions. Novel risk-factors associated with respiratory diseases were also identified.

## Introduction

Acute and chronic respiratory diseases contribute to significant morbidity and mortality across the world, more so in the developing countries. The global burden of disease (GBD) reports have highlighted a high burden of chronic respiratory diseases (CRDs) in India [1, 2]. In an earlier study from India it has been reported that over half of patients who visited primary care physicians across 880 cities and towns, did so for respiratory symptoms [3]. Factors such as air pollution and tobacco use may explain high burden due to respiratory diseases only partly. Apart from the association of traditional risk factors, seasonal exacerbations of asthma and COPD have been evaluated in some studies. Asthma exacerbations have been reported in September in school going children in the western world [4, 5]. In a retrospective cohort study based on database of general practitioners taking care of Dutch population, seasonal effects were observed in the incidence of asthma [6]. Similarly, COPD related morbidity has been shown to be more in winters as compared to summer season in a retrospective analysis of pooled data (1.65-fold and 1.56 fold higher exacerbations in winters in the northern and southern hemisphere respectively) [7].

Seasonal variations in clinical presentation of other respiratory disorders have not yet been explored comprehensively in large scale prospective studies.

The Indian Meteorology Department (IMD) identifies four climatological seasons in India, viz: winter (December to February), summer or pre-monsoon (March to May), monsoon or rainy season (June to September) and post-monsoon or autumn (October to November). Monsoon rains arrive usually from June to September in India and this period can be considered as a wet season as against dry season prevailing over the rest of the year [8].

In the Seasonal waves of respiratory disorders (SWORD) study, we evaluated the proportionate clinical burden of different respiratory diseases presenting to outpatient clinics of physicians across India on four seasonal transitions.

## Methods

The SWORD study was a multicenter, point-prevalence study of symptomatic patients presenting in outpatient clinics across India on four different days of the year representing seasonal transitions viz: winter, summer, monsoon or rainy season and autumn. This study was conceived and co-ordinated at Asthma Bhavan, an independent research institute in Jaipur, Rajasthan.

Sampling Frame: Respiratory physicians including pulmonologists and internal medicine specialists in India registered under the leading chest societies of country such as Indian Chest society (ICS), National College of Chest Physician (NCCP), and participants of the National Pulmonary Conference (NAPCON) 2015 were invited to participate in the survey. An advertisement was also published in the ICS journal Lung India, inviting physicians and pulmonologists to participate in the survey. At least two communications through postal letter, email, text message and phone were sent to all these chest physicians.

All the participating physicians were connected through social media. Online training for the standard operating procedure of the survey, filling up of proforma and logistics of the study was given. Frequently asked questions were also discussed and addressed in the group. Subsequently, this media was used for providing information as well as for training and awareness before the study.

The study was conducted in four seasonal phases during year 2017–18 to report burden of various symptomatic respiratory diseases in outpatient department of physicians involved in respiratory practice. Climatological seasons included winter (December to February), summer (March to May), monsoon or rainy season (June to September) and post monsoon or autumn (October to November) as per IMD [7].

Point prevalence dates were predetermined for first week of February, May, August and November. However, holiday break and a day after the holiday were excluded in order to avoid unequal distribution of outpatient load during those days.

The study proforma was developed after extensive literature search on PubMed and Cochrane database. ICD-10 document on respiratory diseases, GINA asthma, GOLD COPD and documents available on chronic respiratory diseases were consulted. The proforma included the preliminary information regarding site investigator, and the study questionnaire. Demographic data, risk factors, comorbidites, and all ICD-10 based respiratory diagnoses were included in the proforma. Relevant investigations and treatment information were also included. The questionnaire was initially pilot tested by 15 randomly selected physicians from two cities in India. Minor logistical issues identified during initial pilot survey were rectified. The questionnaire was found to be easy to fill and required not more than 5 min to record data of a single patient. An online format of the same questionnaire did not fare well in subsequent pilot testing and therefore the print format was finalized.

On the study day, all the consecutive patients were given an identifier number on the OPD slip and the final diagnoses was entered in proforma by participating physician after history, clinical examination and relevant investigations. Verbal informed consent form and instructions for taking consent were provided to the site investigators. The study did not include minors.

Patients attending outpatient services with respiratory conditions were included. Only those sites were included who completed survey on all four seasonal days.

For descriptive purpose, the country was divided into 5 parts: north, south, east, west, and central region. Northern part included states of Punjab, Haryana, Himachal Pradesh, Jammu Kashmir, Chandigarh and Delhi. Western part included Rajasthan, Maharashtra, Gujrat and Goa. Southern part included Karnataka, Andhrapradesh, Telangana, Tamilnadu, Pondichery and Kerala. Eastern part included Bihar, Jharkhand, Orisa and West Bengal, Assam, Manipur, Tripura, and Arunachal Pradesh, and Central region included Madhya Pradesh, Uttar Pradesh, Uttrakhand, and Chhatisgarh.

The relationship of prevalence of respiratory diseases with the human development index (HDI) was assessed. HDI is a composite index and includes life expectancy (health dimension), education (knowledge dimension) and per capita gross national income (standards of living) [9]. It was classified as higher >+ 0.704, middle 0.640–0.703, and lower <0.639 as per the United Nations development program 2018. The Indian national average HDI was 0.639 during the study period. India State Level Human Development Index is available at the government of India and Niti Aayog websites. We used the state-level HDI in our study.

The detail of methodology of this study protocol has been published previously [10]. The study protocol was approved by ethics committee of the coordinating centre prior to commencement of this prospective multi-centre observational study (IECAB/2017/85).

### Data management

The data were collected in the paper format. All the PI’s were asked to enter data in the hard copy and send the same to the SWORD team at national coordinating centre. The format of study questionnaire allowed to hide the identity of patient automatically. Information regarding the study sites was also entered anonymously with help of coding.

The forms were then arranged in order and checked for completeness of the data. The data entry operators were trained for double data entry. The data were then entered in an Excel sheet by the team of four data entry operators. Double data entry technique was adopted to minimize the entry errors. The data manager checked 10% of the entered forms daily as a part of quality check. Any discrepancy found in the quality check was resolved. After completing the data entry for all the sites data were transferred to software, cleaned and subsequently locked.

### Statistical analysis

The demographic and clinical characteristics are summarized using means and standard deviations (SD) for continuous variables and count and percentages for categorical variables. Comparison of continuous variables was done with t-tests and ANOVA test while categorical variables were analysed using the χ2 test. The odds ratios (OR) and 95% CIs for association of risk factor with different conditions were computed by simple binary logistic regression. We performed multivariable logistic regression adjusted for the cluster of risk factors and for comorbid condition separately. Seasonality of different respiratory diseases was assessed using χ2 trend analysis. All analyses were performed using commercially available software (SPSS, Version 22.0, SPSS Inc., Chicago) and a P value of <0.05 was considered significant at 95% confidence intervals.

## Results

Initially, of the 4108 qualified physicians contacted, 420 volunteered to participate in the study. A total of 366 sites contributed to the study in the first phase in August 2017 followed by 348 sites in the month of November of the same year representing monsoon and autumn phases respectively. Winter and summer phases took place in February at 336 sites and May 2018 at 318 sites, respectively but for convenience of description we will hereafter express contiguous yearly phases as winter, summer, monsoon and autumn. A total of 29,266 patients attended respiratory outpatient department on four study phases at study sites distributed across the country.

**Fig 1:**
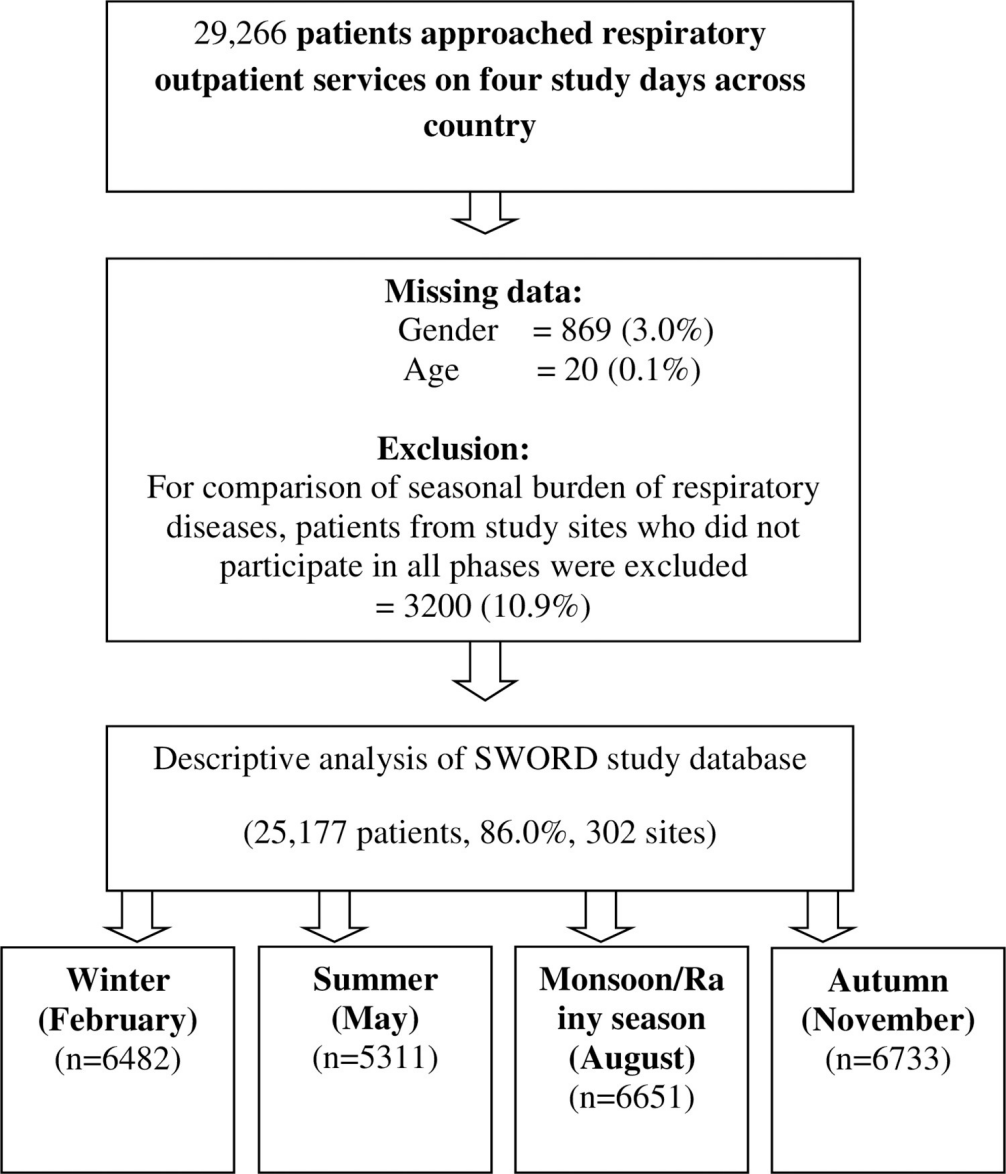
Flow diagram. A total of 302 sites across the country were included for assessment of seasonal patterns in the SWORD survey. The flow diagram shows the selection of sites and division of study population according to seasonal phases.

However, for assessment of effect of seasonal transitions on outpatient attendance of patients with respiratory diseases, we included only those sites who participated on all four seasonal days. Therefore, data from physicians not having participated in all phases (3200, 10.9% patients) were excluded from assessment. Finally, 25177 (86.0%) patients from 302 centres participated in all phases of study and underwent comparative analysis for seasonal symptomatic burden **(Fig 1)**.

Out of 36 states and union territories of India, 32 were covered in the SWORD study. From the northern region 86 (28.5), southern region 66 (21.8), eastern region 56 (18.5), western region 40 (13.2), and central region 54 (17.9) sites participated.

Out of 25177 patients from 302 centres across India, 14102 (56.0%) were males and 11075 (44.0%) were females. There was male preponderance in all geographic regions of India with the highest 58.0% in eastern region and the lowest 53.0% in southern region. There were a total of 155 (1.4%) pregnant females. The mean age of study population was 46.1±18.1 years. There were 8055 (32.0%) patients attending outdoor clinics at public healthcare services and 17122 (68.0%) patients attending private healthcare services (Table 1).

**Table 1:** Number of patients, demographic factors, healthcare settings and presenting symptoms.

Parameter	Total study population	Winter (February)	Summer (May)	Monsoon (August)	Autumn (November)	P value
Number of patients	**(N = 25177)**	**(N = 6482)**	**(N = 5311)**	**(N = 6651)**	**(N = 6733)**	
Number of centers	302	302	302	302	302	
Number of physicians	308	308	308	308	308	
Male	14102(56.0)	3673(56.7)	3021(56.9)	3640(54.7)	3768(56.0)	0.066
Female	11075(44.0)	2809(43.3)	2290(43.1)	3011(45.3)	2965(44.0)	
Age≤ 40 yr	10383(41.2)	2615(40.3)	2192(41.3)	2683(40.3)	2893(43.0)	0.019
Age 41–60 yr	8540(33.9)	2200(33.9)	1787(33.6)	2307(34.7)	2246(33.7)	
Age >60 yr	6254(24.8)	1667(25.7)	1332(25.1)	1661(25.0)	1594(23.7)	
BPL*	3803(15.1)	937(14.5)	755(14.2)	1150(17.3)	961(14.3)	0.514
Pregnancy	155(1.4)	43(1.5)	25(1.1)	47(1.6)	40(1.3)	0.467
Public sector	8055(32.0)	2094(32.3)	1654(31.1)	2190(32.9)	2117(31.4)	0.129
Private sector	17122(68.0)	4388(67.7)	3657(68.9)	4461(67.1)	4616(68.6)	

*BPL = below poverty line- India

It includes seasonal distribution of proportionate patient load, socio-demographic profile, and health-care settings in the SWORD study.

A total of 3803 (15.1%) patients belonged to below poverty line (BPL) families. A total of 2539 (10.1%) patients were asymptomatic (Supporting information section) and revisits were 14692(58.4%).

Among respiratory symptoms, breathlessness (15287, 60.7) was the most common complaint.

**Fig 2:**
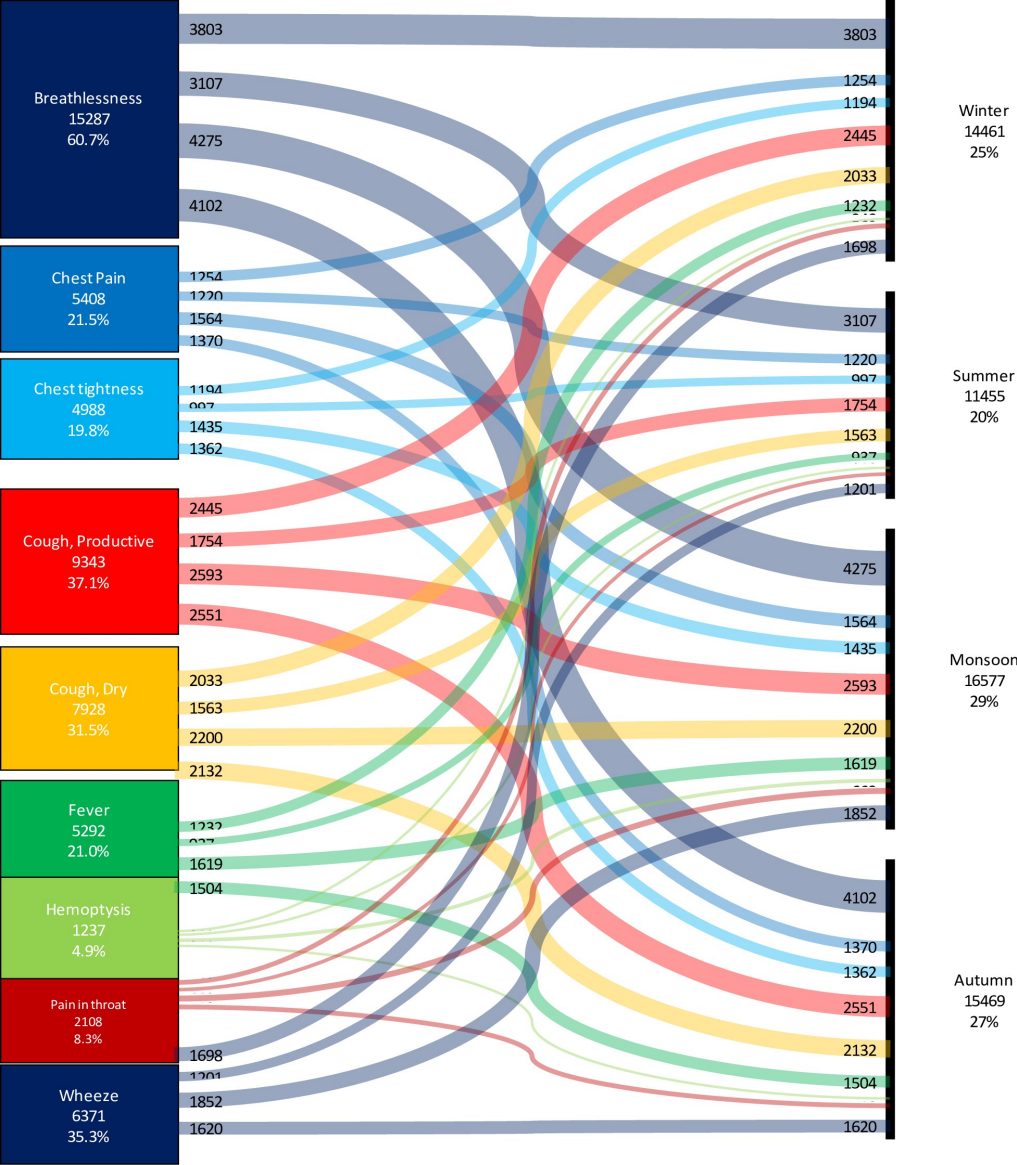
Sankey graph of symptoms due to respiratory disorders in the sword study across the seasons. Cough (17266,68.6%) breathlessness (15287,60.7%), and wheeze (6371,35.3%) were the most common symptoms. Symptom load was relatively less (11455,20%) during summer season.

Productive cough (9343, 37.11%), dry cough (7928, 31.5%), wheeze (6371, 25.3%), chest pain (5408, 21.5%) and chest tightness (4988, 19.8%) were the other common symptoms. There were 4481 (17.8) patients with one symptom, 7297 (29.0) patients with two symptoms, 6260 (24.9) patients with three symptoms and 4600 (18.2) with more than 3 symptoms (Fig 2).

Asthma (7505, 29.8%), and COPD (3916, 15.6%) were the most common diagnoses in the study.

**Fig 3:**
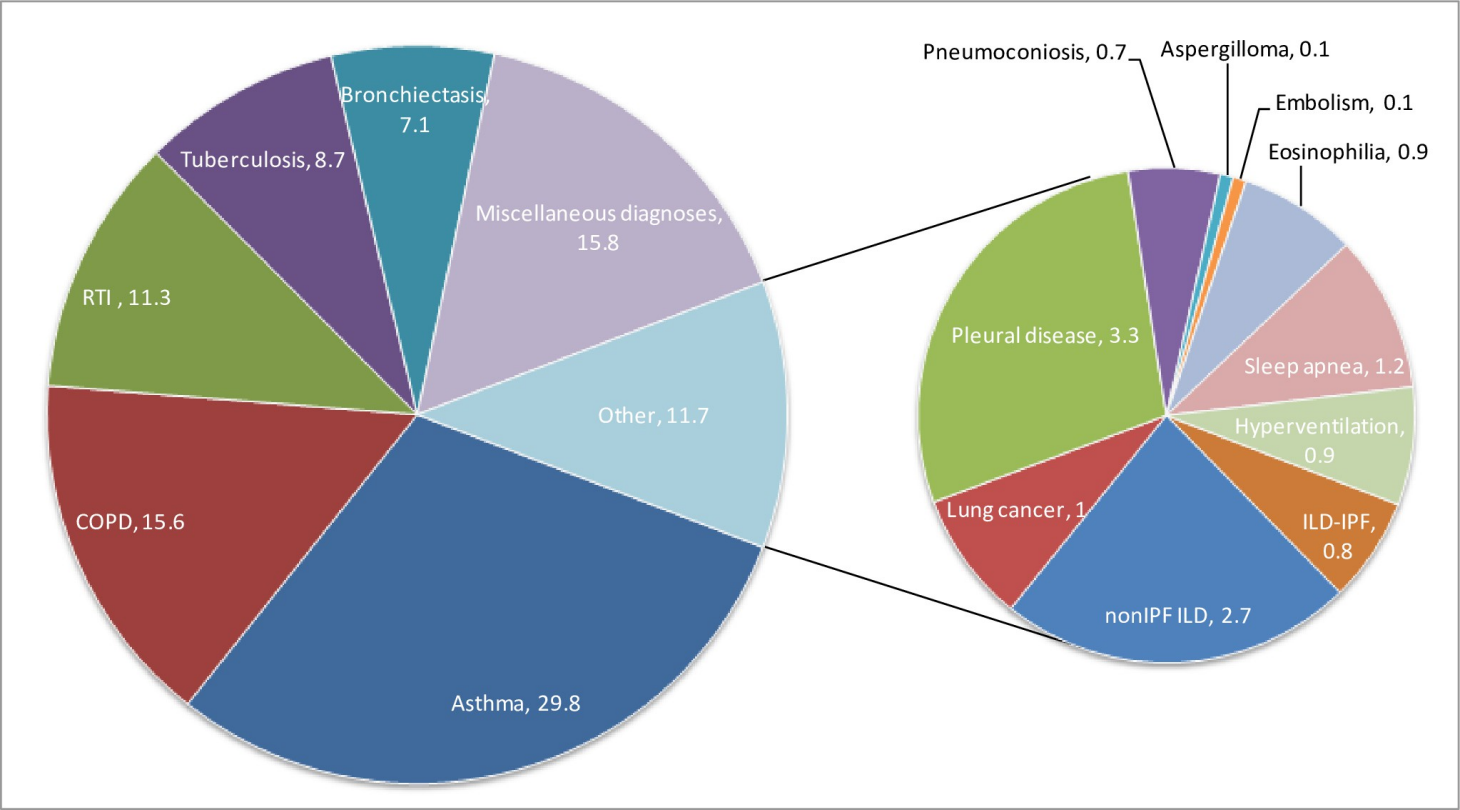
Respiratory diagnoses in the SWORD study population. Asthma, COPD, RTI, tuberculosis and bronchiectasis were the major respiratory diagnoses. As per the SWORD study protocol all the ICD-10 based respiratory diagnoses were included. Other miscellaneous respiratory diagnoses are not shown. (Abbreviations: COPD = chronic obstructive pulmonary disease, HVS = hyperventilation syndrome, RTI = respiratory tract infections, IPF = idiopathic pulmonary fibrosis).

Respiratory tract infections (RTI including upper respiratory infections and pneumonia; 2851, 11.3%), tuberculosis (2196, 8.7%), and bronchiectasis (1799, 7.1%) were the other common diagnoses. (Fig 3).

### Asthma

The mean age of asthma patients was 42.8±17.5 years.

**Fig 4:**
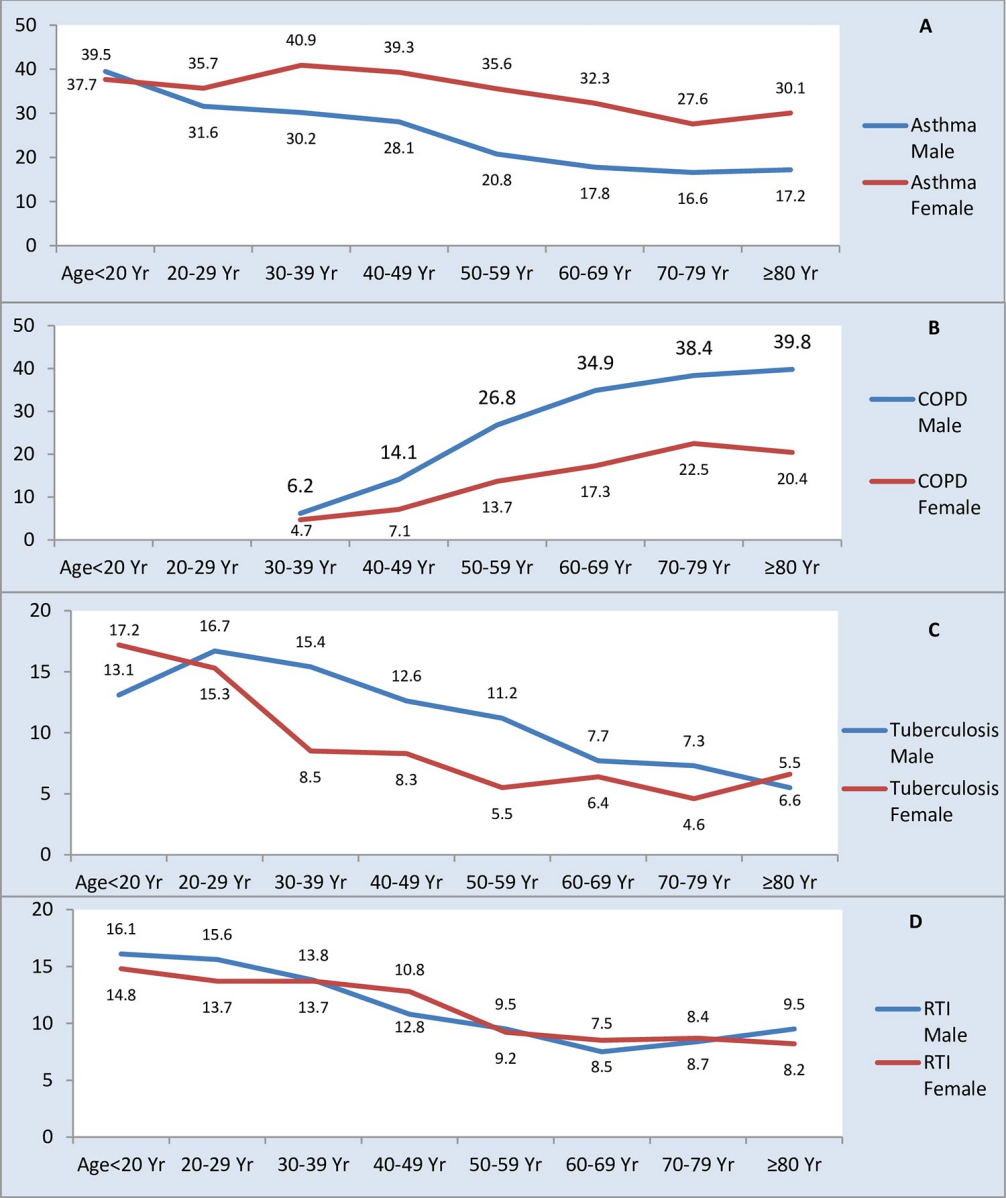
Age-wise proportion of respiratory diseases in males and females in the study population. Age-and sex wise distribution of prevalence of the major respiratory diseases. Differing distribution by age-and sex group were seen. Y-axis shows proportion of cases in a gender as per the age-groups specified.

The prevalence of asthma decreased as the age advanced in males while a mild rise was noted at 30–49 year and more than 80 year in females (Fig 4A).

**Fig 5:**
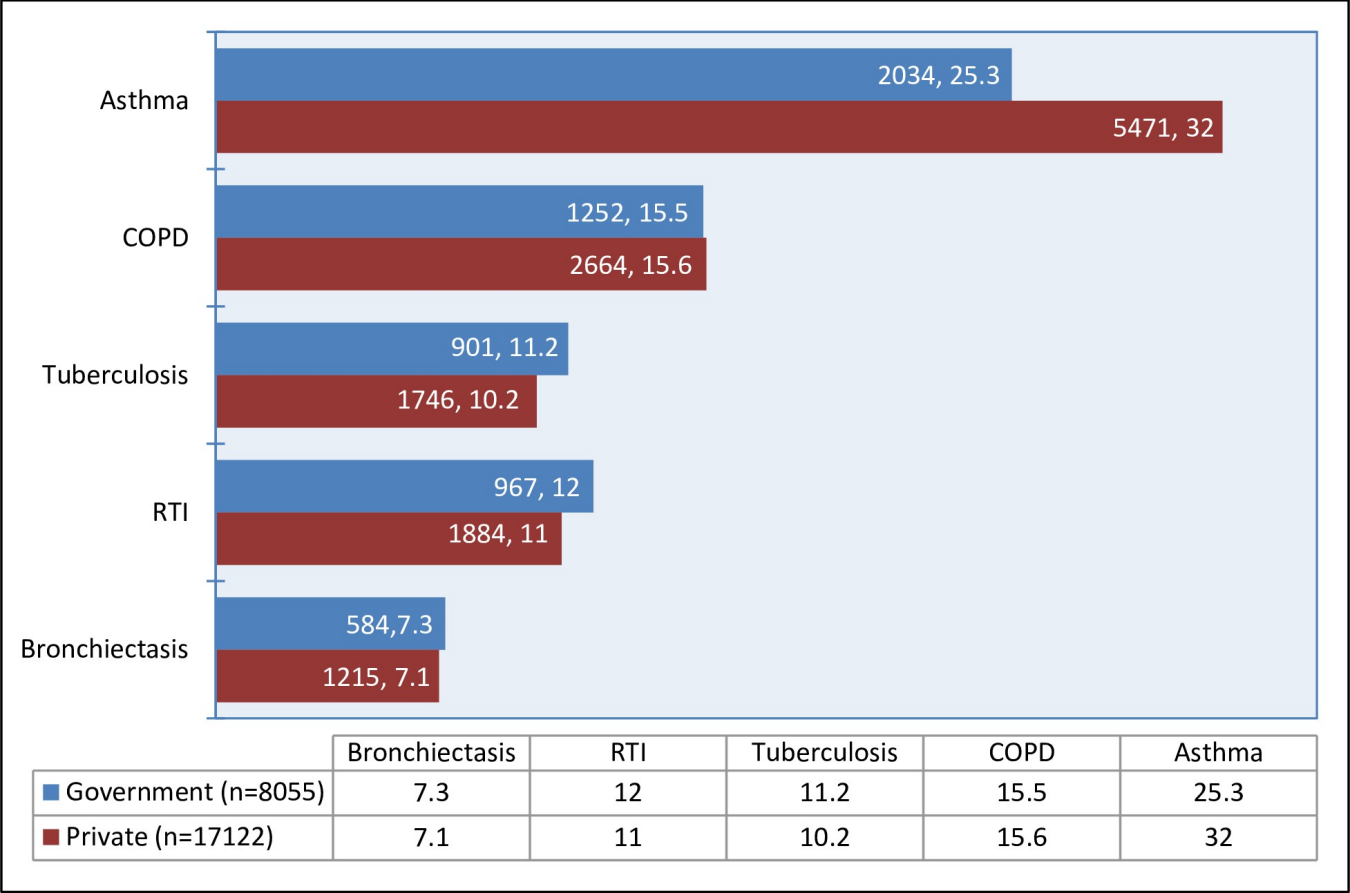
Clinical care settings vs. respiratory diagnoses in the SWORD study. Patients presenting in the public vs. private sector as per the major diagnoses. Asthma was seen more commonly at private settings (p<0.001) while RTIs and tuberculosis in the public healthcare services (p<0.05). COPD and bronchiectasis did not show significant difference (p>0.05).

Asthma was seen more frequently at private hospitals (5471, 32.0% vs. 2034, 25.3%; p<0.001) (Fig 5).

**Fig 6:**
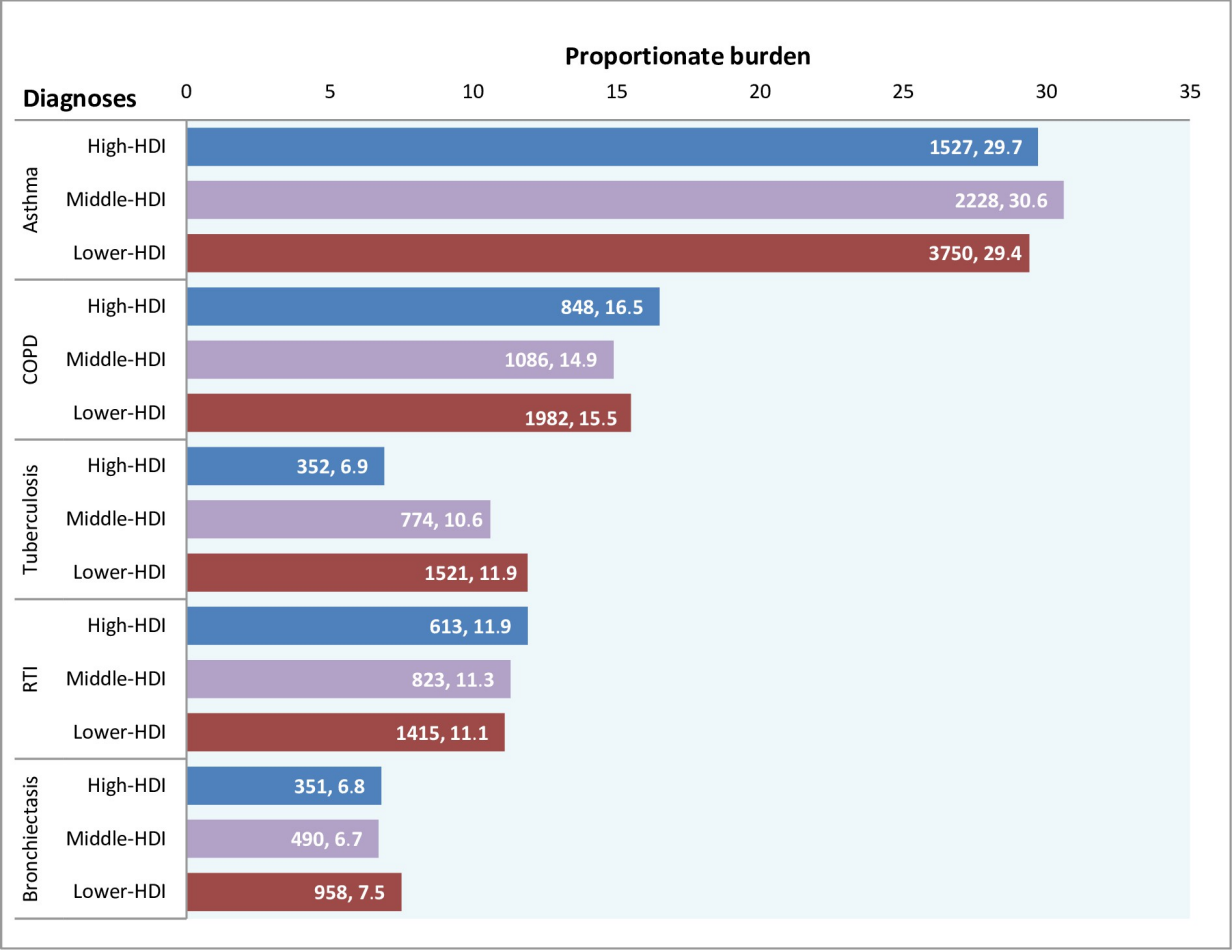
Human development index (HDI) versus respiratory diagnoses in the SWORD study. Human development index (HDI) stratification as per the respiratory disease diagnosis. Tuberculosis was more common in lower HDI (p<0.001) and RTIs in the middle HDI (p<0.18). Distribution of asthma, COPD, and bronchiectasis were not significantly affected by HDI strata (p>0.05).

There was no significant effect of HDI strata on occurrence of asthma (1527,29.7% vs. 2228,30.6% vs. 3750,29.4%;p>0.05) (Fig 6).

**Fig 7:**
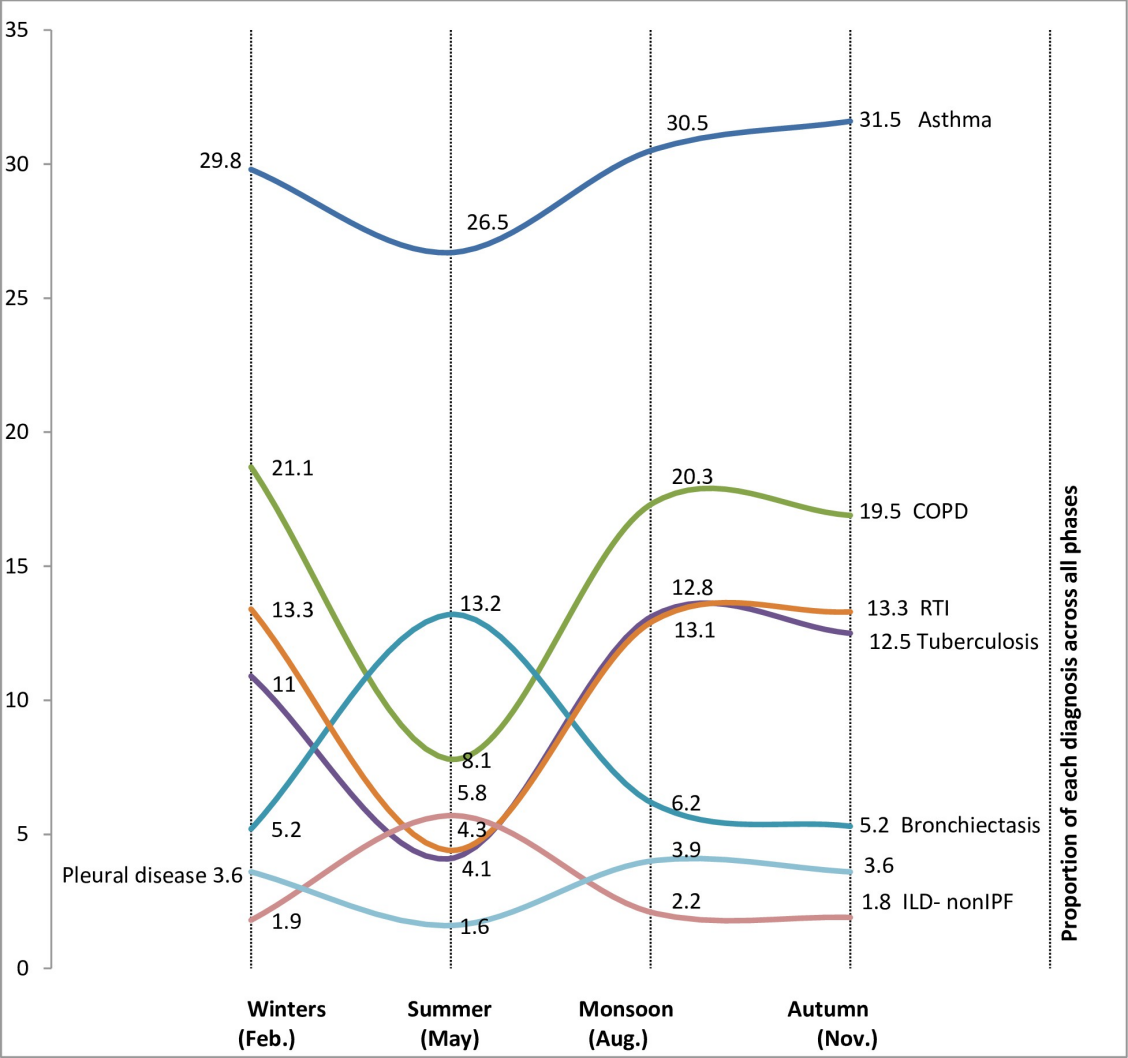
Seasonal patterns of major respiratory diseases in the SWORD study. Seasonal variations in the proportionate distribution of respiratory diseases at respiratory services across India. All major diseases including asthma, COPD, tuberculosis, RTIs, and bronchiectasis showed significant seasonal fluctuations. (Abbreviations: COPD = chronic obstructive pulmonary disease, RTI = respiratory tract infections including pneumonia, ILD = interstitial Lung Disease, IPF = idiopathic pulmonary fibrosis).

Asthma showed significant seasonal variations (Fig 7, peak 31.5% autumn vs. trough 26.5% summer; p<0.001).

**Fig 8:**
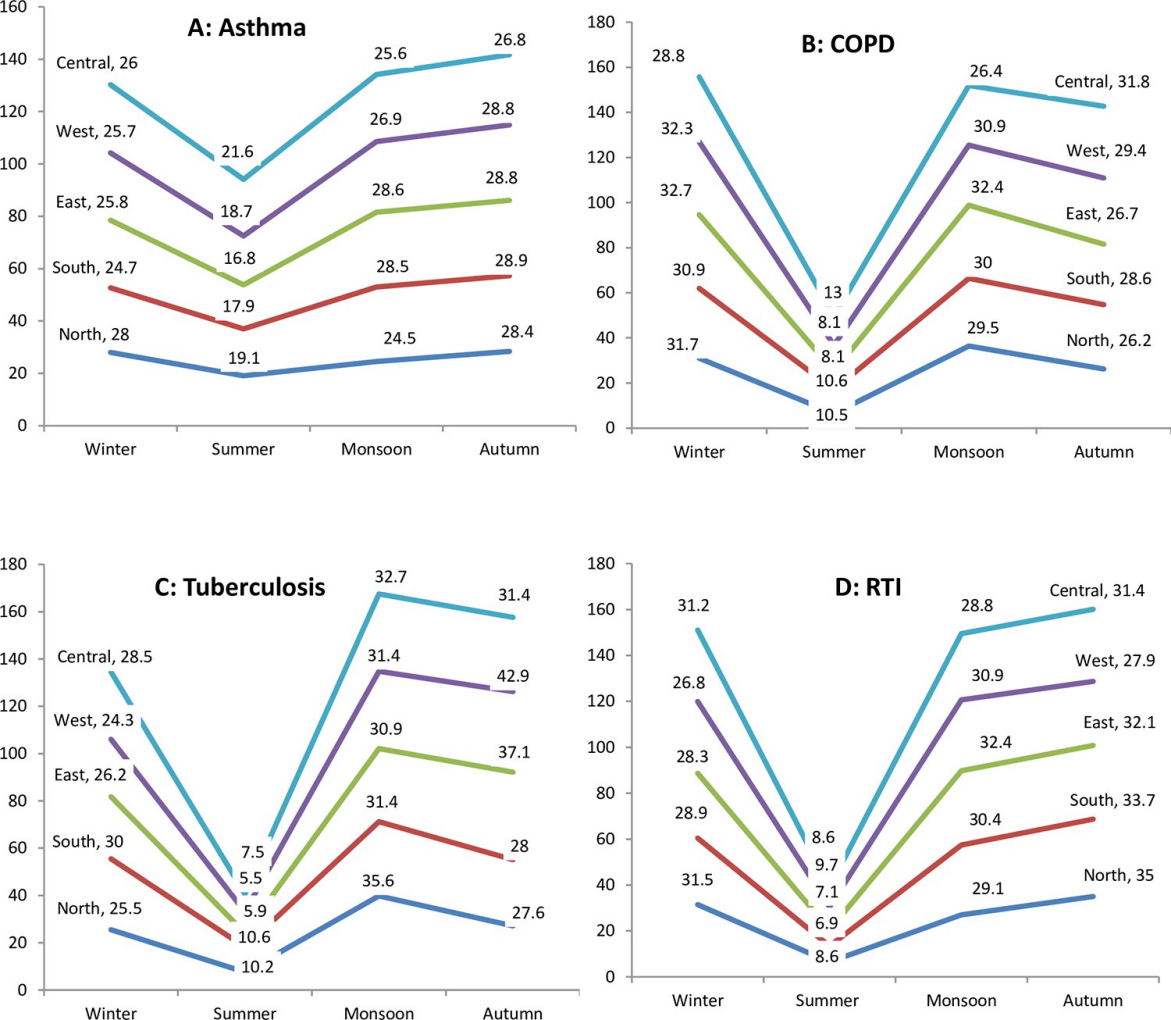
Seasonal patterns of respiratory diagnoses in major geographic zones of India. Regional distribution of seasonal trends for asthma, COPD, tuberculosis, and respiratory tract infections (RTIs), in the north, south, east, west and central regions respectively. The height of the boxes represents proportionate clinical burden and the ribbon over the top of boxes represents seasonal fluctuations. Summer dip are noticeable for all major respiratory diagnoses across the regions (p value<0.001).

All the regions across India showed a dip in symptomatic asthma in the summers and subsequent rise pattern. The peaks were seen in autumn in all regions except western region where peak was seen in winters (Fig 8A).

Asthma was associated with bird and pets at home (OR:1.07, CI:1.01–1.14), visible mold exposure at home and work place (OR:1.12, CI:1.03–1.22), recent travel (OR:1.22, CI:1.13–1.32), and rain-wetting (OR:1.27, CI:1.15–1.40) (Table 2). The question included in the study proforma for mold exposure was “Visible mold at home /work place” and for rain wetting was “got wet in rain during last 1week.” (Supporting information section).

**Table 2:** Association of the major respiratory diagnoses with risk factors and co-morbid conditions.

Risk Factors	Asthma	COPD	Tuberculosis	RTI
Biomass fuel exposure	0.98(0.92–1.04)	**1.42(1.31–1.54)**	1.02(0.93–1.12)	0.82(0.75–0.91)
Birds/Pets at home	**1.07(1.01–1.14)**	0.94(0.86–1.02)	0.89(0.80–0.98)	1.03(0.93–1.13)
Rain-wetting	**1.27(1.15–1.40)**	**0.76(0.66–0.87)**	0.87(0.75–1.02)	**1.53(1.34–1.74)**
Smoker/Ex-smoker	**0.42(0.39–0.45)**	**4.93(4.58–5.29)**	1.01(0.92–1.10)	0.67(0.61–0.74)
Visible mold exposure	**1.12(1.03–1.22)**	1.04(0.93–1.17)	1.07(0.94–1.22)	0.84(0.74–0.96)
Work in mine	0.88(0.76–1.02)	0.95(0.81–1.12)	**1.27(1.06–1.53)**	0.75(0.60–0.94)
Recent travel	**1.22(1.13–1.32)**	**0.74(0.67–0.83)**	0.90(0.79–1.01)	**1.17(1.05–1.30)**
**Co-morbid condition**	**Asthma**	**COPD**	**Tuberculosis**	**RTI**
Allergic diatheses*	**3.93(3.70–4.16)**	0.44(0.40–0.48)	**0.28(0.24–0.31)**	1.05(0.97–1.15)
Anaemia	0.76(0.67–0.86)	0.88(0.76–1.02)	**1.95(1.69–2.25)**	**1.21(1.03–1.41)**
Arthritis	0.95(0.82–1.09)	**1.45(1.25–1.68)**	0.71(0.56–0.90)	0.97(0.80–1.18)
Diabetes	0.94(0.84–1.04)	**1.20(1.08–1.34)**	**1.26(1.10–1.45)**	0.95(0.83–1.10)
GERD	0.89(0.82–0.97)	**1.31(1.18–1.44)**	0.83(0.72–0.95)	0.92(0.82–1.04)
Hypertension	1.02(0.93–1.10)	**1.93(1.77–2.11)**	**0.34(0.29–0.40)**	0.85(0.76–0.96)
CAD	0.83(0.71–0.96)	**1.80(1.56–2.07)**	**0.50(0.38–0.69)**	1.10(0.91–1.34)

*(Allergic diatheses = Rhinitis/Urticaria/Eczema)

Shows relationship of major respiratory diagnoses with risk factors and co-morbidities in the SWORD study. Adjusted odds ratios calculated by binary logistic regression are shown followed by their 95% confidence intervals (CIs). The significant odds ratios are highlighted in bold letters. Odds ratios were internally adjusted for risk factors or co-morbid conditions within the group e.g., biomass adjusted with birds/pets at home, rain-wetting, smoking, visible mold at home or work place, work in mine, recent travel.

Asthma was also strongly associated with allergic diatheses including allergic rhinitis, urticaria and eczema (OR:3.93,CI:3.70–4.16).

### COPD

The mean age of COPD patients was 58.9±13.8 years. It was more common in the males 2853 (20.2) as compared to females 1063 (9.6) (p<0.001). Prevalence of COPD increased with age in both sexes which was more marked after the age of 40 years where COPD was almost twice as common in males as in females (Fig 4B). COPD was seen with equal frequency at private services as well as public sector (2664,15.6% vs. 1252,15.5%; p<0.5) and there was no significant effect of HDI (848,16.5 vs. 1086,14.9 vs. 1982,15.5; p>0.05) (Figs 5 and 6). COPD also showed seasonal variation (peak 21.1% winter vs. trough 8.1% summer; p<0.001) (Fig 7), the peaks were seen in winters in all except central regions where peak was seen in autumn (Fig 8B). COPD was significantly associated with biomass fuel exposure (OR: 1.42, CI:1.31–1.54) and smoking (OR:4.93, CI:4.58–5.29). It was also associated with co-morbidities like arthritis (OR:1.45, CI:1.25–1.68), diabetes (OR:1.20, CI:1.08–1.34), gastro-esophageal reflux disease (GERD) (OR:1.31, CI:1.18–1.44), hypertension (OR:1.93, CI:1.77–2.11) and coronary artery disease (OR:1.80, CI:1.56–2.07) (Table 2).

### Tuberculosis

The mean age of tuberculosis patients was 40.5±17.4 years. Tuberculosis was more common in males especially in 30–49 years except at extremes of ages (Fig 4C). It was more common in the males 1640 (11.8%) as compared to females 1007 (9.1%). It was seen more frequently in the government healthcare services (901,11.2% vs. 1746, 10.2%; p<0.02) (Fig 5). Tuberculosis was also associated with poor socio-economic status as the difference between upper, middle and lower HDI was statistically significant (352, 6.9% vs. 774, 10.6% vs. 1521,11.9% respectively; p<0.001) (Fig 6). Seasonal variation in presentation existed for tuberculosis (peak 13.1% autumn vs. nadir 4.1% summer; p<0.001). Summer dip patterns were relatively more prominent for tuberculosis in all regions and it showed peaks in monsoon and autumn (Fig 8). The associated factors included stone mining (OR:1.27, CI:1.06–1.53), anaemia (OR:1.95, CI:1.69–2.25), and diabetes (OR:1.26, CI:1.10–1.45) (Table 2).

### RTI

The mean age of RTI patents was 41.9±17.8 years. RTI did not show any significant difference in prevalence with respect to age or gender (Fig 4D). The presentation was more common in the public sector versus private sector (967, 12.0% vs. 1884, 11.0%; p<0.02) (Fig 6). RTIs had significant seasonal variations (peak 13.3% winter and autumn vs. nadir 4.3% summer; p<0.001) (Fig 7); a summer decline was seen followed by rise and minor fluctuations in other seasons in all the regions(Fig 8D). RTIs were associated with rain wetting (OR:1.53, CI:1.34–1.74), recent travel (OR:1.17, CI:1.05–1.30) and anaemia (OR:1.21, CI:1.03–1.41) (Table 2).

### Bronchiectasis

The mean age of bronchiectasis patients was 48.7±17.1 years and did not show any significant difference in prevalence with respect to age, gender or healthcare sector. Bronchiectasis (nadir 5.2% winter and autumn vs. peak 13.2% summer; p<0.001) (Fig 6) also had seasonal variation in presentation. Bronchiectasis was associated with biomass fuel exposure (OR:1.38, CI:1.24–1.53) and smoking (OR:1.17, CI:1.05–1.30).

### Other diseases

Asthma-COPD overlap (1430, 5.7%), pleural diseases (824, 3.3%), post-tuberculosis COPD (686, 2.7%), non-IPF ILDs (685, 2.7%), IPF (214, 0.8%), lung cancer (248, 1.0%), sleep apnoea (297, 1.2%), hyperventilation syndrome (220, 0.9%), pneumoconioses including silicosis, asbestosis, and coal workers pneumoconiosis (173, 0.7%), pulmonary eosinophilia (216, 0.9%), aspergilloma (34,0.1%), and pulmonary embolism (34,0.1%) were comparatively rare. Pneumoconioses were associated with lower HDI (p = 0.034).

Pleural disease (peak 3.9% monsoon vs. trough 1.6% summer; p<0.001) showed clear nadirs in summer season (Fig 7). In contrast, and non-IPF ILD (nadir 1.9% winters vs. peak 5.8% summer; p<0.001) showed a rise in the summer season. Lung cancer and IPF did not show any observable seasonal fluctuations (not shown in the figure).

## Discussion

This is one of the largest, multicentre studies analysing the seasonal effects on the presentation of respiratory conditions conducted on four different days of a year across 302 sites covering 25,177 adults in India. In this study, the most common causes of visit to OPD were asthma (29.8%), COPD (15.6%), RTI including pneumonia (11.3%), tuberculosis (8.7%), and bronchiectasis (7.1%). Significant seasonal variations were present in all major respiratory diseases namely asthma: 31.5% autumn vs. 26.5% summer, COPD: 21.1% winter vs. 8.1% summer, RTIs: 13.3% winter vs. 4.1% summer, and tuberculosis: 13.1% monsoon vs. 4.1% summer (p<0.001 for each condition respectively). Furthermore significant association was found with trigger factors for each respiratory disease viz. exposure to molds, household animals, recent-travel, and rain-wetting for asthma; biomass fuel and smoking for COPD; and rain-wetting and recent-travel for RTIs. The association of bronchial asthma visits with rain-wetting and travel may be related to factors like acute exacerbation of bronchial asthma or viral infections. Data on viral infections were not recorded. Probably, further studies are required to validate this notion. Similarly, higher age could be a contributory factor for association of COPD with many comorbid illnesses.

Most of the respiratory disorders in the SWORD study showed seasonal variations. Asthma, COPD, tuberculosis as well as RTIs showed dip in amplitude mainly during summer. Lesser prevalence of symptomatic respiratory patients during summer in the SWORD study is intriguing and could be partly explained by environmental factors including rise in ambient temperature, and prolonged, overhead and intense sun light throughout the days, less vegetation and molds. A retrospective analysis of global electronic search data has reported a high seasonal rhythm for chronic diseases with inverse hemispheric patterns [11]. Amplitude and duration of a seasonal curve are the main factors that define seasonality. RTIs like influenza may have up to 60% annual oscillations [12]. Seasonal variation mainly depend on temperature, precipitation and the productivity of the biosphere [13].

Another possible hypothesis to explain the seasonal variation in respiratory disease is vitamin D deficiency. It should be noted that acute change in vitamin D may not affect the point prevalence of a disease. However, trends may be noted after sustained exposure. Seasonality of respiratory diseases especially tuberculosis has been explored in detail in past. In a systematic review of peer reviewed studies from 11 countries across the world, it was found that vitamin D variability, change in immune function, and delay in diagnosis of disease were the main factors for seasonal tubercular disease [14]. The role of vitamin D has been proposed in the pathogenesis of COPD as well. In a systematic review and meta-analysis, vitamin D deficiency was significantly associated with increased risk of COPD and severe COPD, [15]. Based on this concept, ample sunlight in the summers would thus, lead to vitamin D synthesis and may reduce the prevalence of CRDs in the summers.

The latter part of summer and beginning of monsoons is marked with rains, fluctuations in temperature, changes in flora and fauna. These may be responsible for the rise in respiratory illnesses during this period. The high prevalence of respiratory diseases related outpatient visits is maintained subsequently in winters probably due to rise in pollution or mixed effect of viral related illnesses [16]. In a large prospective cohort of elderly people from Hong Kong, wintertime temperature variability was associated with significant risk of hospitalizations due to pneumonia and COPD, with hazard ratios: 1.15(1.01–1.31) and 1.41(1.15–1.71), respectively while no such effect was observed during summertime [17].

In contrast to the prevalence of asthma, COPD and tuberculosis, the seasonal patterns of non-IPF ILD, and bronchiectasis in SWORD study showed an increase in summer; which would be difficult to explain and would be a matter of research. Exacerbations of these diseases are linked to common infections and summer seasonal patterns are reported with some respiratory viruses especially human rhinoviruses [18]. Seasonal patterns were also observed in different geographic regions of India. A decreased disease burden of respiratory diseases was consistently seen in summer season in almost all regions of India.

The SWORD study showed that 45.4% of outpatients presenting to Indian respiratory services were asthma and COPD. The point prevalence POSEIDON study, conducted on a single day to capture outpatient data from general practitioners showed significant burden of patients with respiratory symptoms (50.6%) [3]. Almost 15% of the entire patient pool presenting to the outpatient clinic of general practitioners participating in the POSEIDON study were due to obstructive airway disease. In a similar site-based study on patients presenting with diagnosis of respiratory disease during a single visit (APBOARD study) from Asia-pacific region, asthma was the most frequent primary diagnosis followed by allergic rhinitis, COPD, and rhinosinusitis [19].

Public and private OPDs are the two types of outpatient services provided to patients in India. Public OPDs are cheap and cater patient with lower social strata. Private OPDs are expensive and the cost of consultation, investigations and medications has to be borne by the patient. Patients with asthma presented more frequently at private OPDs (p<0.001) and tuberculosis and RTIs were seen more frequently in the public OPDs (p<0.05). These figures show disparities between states and across social groups, which may prevail in both developing and developed countries. Thus, there is a need for government initiatives to focus on involvement of private sector to achieve the goal of uniform health coverage for all in developing countries [20].

All symptomatic CRD patients particularly tuberculosis in the SWORD study were mainly from lower HDI group (p<0.001). It has been known that social factors such as poor nutrition and overcrowding contribute to the transmission and spread of tuberculosis [21]. In keeping with hygiene hypothesis, asthma was more prevalent in the group with higher and middle HDI though this difference was not statistically significant (p>0.05). On the contrary, the GBD databases showed that increasing sociodemographic index (SDI) led to reduction in DALY due to asthma [22]. GBD analysis used SDI in place of HDI which measures income, education and fertility and the health dimension is not included in SDI.

The major respiratory diagnoses showed specific age-and gender distribution in the SWORD study. Asthma was more common in females while COPD was more common in elderly males. In a large prospective study on Swedish population incident cases of doctor diagnosed asthma were more common in females (OR1.47, 95% CI 1.03–2.09) [23]. According to National Health Interview Survey data 2018, age-adjusted percentage of current asthma was 9.6% in females as against 5.5% in males [24]. Indian GBD data on chronic respiratory diseases showed that the prevalence of asthma was more in females after 20 years age [2]. Biological factors, healthcare seeking behaviour and adherence to treatment may differ between sexes [25]. Similarly, prevalence of COPD increased substantially after 40 years age in SWORD study and reached the highest prevalence in the 75–79 years age group.

The frequency of tuberculosis was more in the 20–59 yrs age with male predilection. RTI was more common in younger age groups and showed a comparable prevalence in both sexes. These findings are consistent with the systematic analysis conducted for the GBD data 1990–2017 including India and other countries, which has documented a predominance of respiratory infections and tuberculosis in younger males [26].

In the SWORD study, proportionate symptomatic burden due to tuberculosis was about 8.7%. This shows that the prevalence of tuberculosis is still high despite implementation of national level tuberculosis programs and programmatic management of drug-resistant TB (PMDT), over the last few years for curtailment of this disease [27]. With this much burden of disease, national goal of eradication of tuberculosis in India by 2025 remains a formidable challenge.

Respiratory tract infections including bacterial and viral pneumonia were seen in almost 11% of study population and also appear to constitute a significant outpatient burden. The relevance of this finding is emphasised by the fact that respiratory infection is usually responsible for aggravation of respiratory diseases leading to significant morbidity, and mortality.

Post infectious bronchiectasis was present in 7.1% of study population. Indian counterpart of the EMBARC bronchiectasis registry has revealed that bronchiectasis in India is more severe than those reported in US and European countries; with a predominance amongst young males. The most common aetiology for bronchiectasis in India is tuberculosis and other respiratory tract infections [28].

Probably, comorbid conditions may increase disease burden of symptomatic patients. Allergic diatheses including allergic rhinitis, urticaria and eczema were strongly associated with asthma in the SWORD study. A large number of comorbid illnesses were associated with COPD including hypertension, coronary artery disease (CAD), diabetes mellitus, GERD, and arthritis. It has also been previously reported that COPD has a high burden of comorbidities which may contribute to worse patient outcomes and mortality [29]. COPD is a multisystem disease and the major etiological factor of COPD is tobacco smoking which also predisposes to other conditions such as CAD. The association of COPD with CAD is important as it may have huge impact on morbidity and mortality due to this disease; further exacerbated by advancing age. Tuberculosis and respiratory tract infections were significantly associated with anaemia. This could be explained by the fact that poor nutritional status predisposes a person to frequent infections including both RTIs and tuberculosis. In a retrospective analysis of healthcare data from India, about half of all cases of active TB among women and slightly more than half of all cases among men were attributable to under-nutrition [30].

Regarding risk factors in the SWORD study, morbidity due to asthma was significantly associated with birds and pets at home, recent travel, mold exposure and rain-wetting. Some of these factors may have served as triggers to an episode of asthma. COPD was significantly associated with smoking, and biomass fuel exposure which is traditionally linked with is disease. Respiratory tract infections were significantly associated with rain wetting and recent history of travel. These risk factors may be responsible for increasing burden of symptomatic patients. The association of anemia with RTI could be due to poor overall health in anemic patients. So far, there is no conclusive evidence in favour of smoking as a risk factor for asthma. A negative association of asthma with smoking in our study is interesting. In the Cracow and Tucson longitudinal epidemiological studies of obstructive airways diseases, a negative association of symptom persistence of medically diagnosed asthma was found in continuous smokers in 19–40 year age category [31]. The lower risk of COPD in rain-wetting and recent travel in our study may be due to the fact that many COPD patients are elderly and immobile and are less likely to leave home especially during rainy days.

The limitations of the SWORD study include lack of simultaneous intercontinental data for comparison of hemispheric patterns. Another limitation is taking point prevalence of single day which may not measure changes in health status in relation to temporal trends of climatic status. We also acknowledge that the variability of temperature and humidity across geographical locations may influence the prevalence of respiratory conditions. Continuous prospective record of seasonal data of a year was not feasible due to multisite nature of the study. Nevertheless, this study covered a large patient population being seen in respiratory outpatient services across India across four days of the year. The uniqueness of this study was record of seasonal patterns of all major respiratory diseases simultaneously across a large geographic area which has not been reported previously.

In conclusion, seasonal patterns in all major respiratory diagnoses were recorded in the present study. Obstructive lung diseases like asthma and COPD are the most common respiratory diseases seen in respiratory outpatient services across India. The seasonal fluctuations of asthma, COPD and tuberculosis were opposite to that noted for non-IPF ILD, and bronchiectasis. There was a strong association of asthma with allergic diatheses while COPD was associated with a large number of comorbid illnesses including CAD. Asthma and RTI were associated with recent travel and rain-wetting. The data pertaining to effect of seasonal influence on burden of respiratory diseases in the country would help in allocating resources as well as in planning further research in this field.

## Supporting information

S1 File Supplementary tables. (PDF)

S2 File (PDF)
