# Supplementary material for: Proportionate clinical burden of respiratory diseases in Indian outdoor services and its relationship with seasonal transitions and risk factors: The results of SWORD survey
Source: PLoS One. 2022 Aug 18;17(8):e0268216. doi: 10.1371/journal.pone.0268216 (PMC9387816; doi:10.1371/journal.pone.0268216)
Supplement: S1 File — (PDF) [file pone.0268216.s001.pdf]

S1 file: Supplementary tables

S1A Table: Composite data

Variables	Total (N=28397)	Male(N=15882)	Female(N=12515)	P Value
Mean age	46.2±18.1	47.7±18.5	44.3±17.3	<0.001
Age≤ 40 years	11624(41.0)	5984(37.7)	5640(45.1)	<0.001
Age; 41-60 years	9671(34.1)	5225(32.9)	4446(35.5)	<0.001
Age; >60 years	7082(25.0)	4658(29.4)	2424(19.4)	<0.001
Below poverty line(BPL)	4257(15.0)	2378(14.9)	1879(15.0)	
Government Hospital	9156(32.2)	5244(33.0)	3912(31.3)	
Private hospital	19241(67.8)	10638(67.0)	8603(68.7)	
**Risk Factors**				
Biomass fuel exposure	7529(26.6)	2970(18.7)	4589(36.7)	<0.001
Birds at home	1820(6.4)	910(5.7)	910(7.3)	<0.001
Pets at home	5809(20.5)	3305(20.8)	2504(20.0)	0.674
Rain-Wetting	2377(8.4)	1336(8.4)	1041(8.3)	0.776
Current smoker	5043(17.8)	4575(28.8)	468(3.7)	0.096
Ex-smoker	3030(10.7)	2604(16.4)	426(3.4)	<0.001
Recent Travel	3929(13.8)	2283(14.4)	1646(13.2)	0.003
Visible mold at home/work	3153(11.1)	1632(10.3)	1521(12.2)	<0.001
Work in mine	1182(4.2)	965(6.1)	217(1.7)	<0.001
**Co morbid Conditions**				
Allergic Rhinitis	8070(28.4)	4023(25.3)	4047(32.3)	<0.001
Anemia	1804(6.4)	669(4.2)	1135(9.1)	<0.001
Arthritis	1349(4.8)	587(3.7)	762(6.1)	<0.001
Diabetes	2960(10.4)	1754(11.0)	1206(9.6)	<0.001
Eczema	503(1.8)	254(1.6)	249(2.0)	0.013
GERD	3630(12.8)	2027(12.8)	1603(12.8)	0.909
Hypertension	4574(16.1)	2550(16.1)	2024(16.2)	<0.001
CAD	1322(4.7)	852(5.4)	470(3.8)	<0.001
Urticaria	602(2.1)	279(1.8)	323(2.6)	<0.001
**Presenting Symptoms**				
Breathlessness	17230(60.7)	9699(61.1)	7531(60.2)	0.126
Chest Pain	6025(21.2)	3578(22.5)	2447(19.6)	<0.001
Chest tightness	5681(20.0)	3187(20.1)	2494(19.9)	0.772
Cough; Productive	10521(37.0)	6410(40.4)	4111(32.8)	<0.001
Cough; Dry	8994(31.7)	4696(29.6)	4298(34.3)	<0.001
Fever	5883(20.7)	3287(20.7)	2596(20.7)	0.987
Hemoptysis	1388(4.9)	900(5.7)	488(3.9)	<0.001
Pain in throat	2384(8.4)	1277(8.0)	1107(8.8)	0.015
Wheeze	7283(25.6)	3973(25.0)	3310(26.4)	0.006
**Present Diagnosis**	**Total (N=28397)**	**Male (N=15882)**	**Female (N=12515)**	**P Value**
Asthma	8510(30.0)	3943(24.8)	4567(36.5)	<0.001
Asthma-COPD overlap	1616 (5.70)	908 (5.7)	708 (5.7)	0.8299

Bronchiectasis- post TB	1504(5.3)	871(5.5)	633(5.1)	0.111
Bronchiectasis-ABPA	743(2.6)	410(2.6)	333(2.7)	0.678
COPD	4512(15.9)	3276(20.6)	1236(9.9)	<0.001
Hypersensitivity pneumonitis	339 (1.2)	194 (1.2)	145 (1.2)	0.628
Hyperventilation syndrome	237(0.8)	116(0.7)	121(1.0)	0.038
ILD-IPF	254(0.9)	126(0.8)	128(1.0)	0.041
ILD- Others	1188(4.2)	679(4.3)	509(4.1)	0.384
ILD- Collagen tissue disease	136(0.5)	45(0.3)	91(0.7)	<0.001
Lung cancer– NSCLC	126(0.4)	88(0.6)	38(0.3)	0.002
Lung cancer– SCLC	64(0.2)	39(0.2)	25(0.2)	0.419
Lung cancer– Other	108(0.4)	70(0.4)	38(0.3)	0.062
Pleural disease– Tubercular	619(2.2)	411(2.6)	208(1.7)	<0.001
Pleural disease– Other	324(1.1)	202(1.3)	122(1.0)	0.019
Pneumoconiosis– Silicosis	118(0.4)	95(0.6)	23(0.2)	<0.001
Pneumoconiosis– Asbestosis	40(0.1)	27(0.2)	13(0.1)	0.140
Pneumoconiosis– Coal worker	23(0.1)	16(0.1)	07(0.1)	0.188
Pneumonia- Bacterial	519(1.8)	298(1.9)	221(1.8)	0.490
Pneumonia- Viral	355(1.3)	195(1.2)	160(1.3)	0.703
Pneumonia- Other	202(0.7)	115(0.7)	87(0.7)	0.773
Post-TB-COPD	755(2.7)	484(3.0)	271(2.2)	<0.001
Pulmonary- Aspergilloma	40(0.1)	31(0.2)	09(0.1)	0.006
Pulmonary-Embolism	40(0.1)	23(0.1)	17(0.1)	0.841
Pulmonary- Eosinophilia	229(0.8)	132(0.8)	97(0.8)	0.600
Sarcoidosis	101(0.4)	50(0.3)	51(0.4)	0.193
Sleep apnea	349(1.2)	231(1.5)	118(0.9)	<0.001
Tuberculosis- Newly diagnosed	1626(5.7)	944(5.9)	682(5.4)	0.075
Tuberculosis- Retreated	566(2.0)	379(2.4)	187(1.5)	<0.001
Tuberculosis-MDR	233(0.8)	138(0.9)	85(0.7)	0.072
URTI	2206(7.8)	1168(7.4)	1038(8.3)	0.003
**Tests**	**Total (N=28397)**	**Male (N=15882)**	**Female (N=12515)**	**P Value**
AFB smear	6111(21.5)	3585(22.5)	2531(20.2)	<0.001
Arterial blood gas analysis	1031(3.6)	618(3.9)	413(3.3)	0.008
Bronchoscopy	687(2.4)	413(2.6)	274(2.2)	0.025
Complete blood count	11621(40.9)	6385(40.2)	5236(41.8)	0.005
CBNAAT	1432(5.0)	858(5.4)	574(4.6)	0.002
Chest X–ray	17285(60.9)	9819(61.8)	7466(59.7)	<0.001
CT of chest	2790(9.8)	1641(10.3)	1149(9.2)	0.001
DLco	417(1.5)	241(1.5)	176(1.4)	0.440
FeNO (Exhaled breath nitric	126(0.4)	57(0.4)	69(0.6)	0.015
oxide)	468(2.9)	338(2.7)	806(2.8)	0.215
Six-minute walk test	295(1.0)	179(1.1)	116(0.9)	0.099
Sleep study	11065(39.0)	6111(38.5)	4954(39.6)	0.058
Spirometry				

S1B Table: Diagnostic tests (seasonal filtered data)

Diagnostic test	Total (N=25177)	Male (N=14102)	Female (N=11075)	P Value
AFB smear	5515(21.9)	3231(22.9)	2284(20.6)	<0.001
Arterial blood gas analysis	868(3.4)	514(3.6)	354(3.2)	0.053
Bronchoscopy	607(2.4)	368(2.6)	239(2.2)	0.020
CBC-blood count	10314(41.0)	5679(40.3)	4635(41.9)	0.011
CBNAAT	1297(5.2)	776(5.5)	521(4.7)	0.004
Chest X-ray	15358(61.0)	8742(62.0)	6616(59.7)	<0.001
CT of chest	2427(9.6)	1431(10.1)	996(9.0)	0.002
DLco	355(1.4)	204(1.4)	151(1.4)	0.578
FeNO	112(0.4)	50(0.4)	62(0.6)	0.015
Six minute walk test	683(2.7)	401(2.8)	282(2.5)	0.149
Sleep study	249(1.0)	155(1.1)	94(0.8)	0.046
Spirometry	9666(38.4)	5365(38.0)	4301(38.8)	0.200

S1C Table: Presenting symptoms (seasonal filtered data)

Symptoms	Total (N=25177)	Male (N=14102)	Female (N=11075)	P Value
Breathlessness	15287(60.7)	8618(61.1)	6669(60.2)	0.149
Chest pain	5408(21.5)	3207(22.7)	2201(19.9)	<0.001
Chest tightness	4988(19.8)	2803(19.9)	2185(19.7)	0.771
Cough- Productive	9343(37.1)	5708(40.5)	3635(32.8)	<0.001
Cough-Dry	7928(31.5)	4173(29.6)	3755(33.9)	<0.001
Fever	5292(21.0)	2972(21.1)	2320(20.9)	0.806
Hemoptysis	1237(4.9)	800(5.7)	437(3.9)	<0.001
Pain in throat	2108(8.4)	1129(8.0)	979(8.8)	0.018
Wheeze	6371(25.3)	3511(24.9)	2860(25.8)	0.093

S1D Table: Distribution of symptoms (seasonal filtered data)

	Total (N=25177)	Male (N=14102)	Female (N=11075)	P Value
Any one	4481(17.8)	2486(17.6)	1995(18.0)	
Any two	7297(29.0)	4118(29.2)	3179(28.7)	
Any three	6260(24.9)	3572(25.3)	2688(24.3)	
> three	4600(18.2)	2631(18.6)	1969(17.7)	
Asymptomatic	2539(10.1)	1295(9.2)	1244(11.2)	

S1E Table: Asymptomatic patients (seasonal filtered data)

		Government(N=8055)	Private(N=12769)	
Asymptomatic		740(9.2)	1799(10.5)	<0.001
	**Higher HDI (N=5136)**	**Middle HDI (N=7272)**	**Lower HDI (N=12769)**	
Asymptomatic	569(11.1)	616(8.5)	1354(10.6)	<0.001

S1F Table: Supplementary table to Figure-4 (seasonal filtered data)

Asthma	**Age<20 Yr**	**20-29 Yr**	**30-39 Yr**	**40-49 Yr**	**50-59 Yr**	**60-69 Yr**	**70-79 Yr**	**≥80 Yr**	**Ptrend**
Total	(N=1716)	(N=3984)	(N=3822)	(N=3987)	(N=4157)	(N=4683)	(N=2253)	(N=575)	
	664(38.7)	1336(33.5)	1357(35.5)	1340(33.6)	1139(27.4)	1091(23.3)	454(20.2)	124(21.6)	<0.001
	(N=926)	(N=2109)	(N=1931)	(N=2017)	(N=2301)	(N=2911)	(N=1528)	(N=379)	
Male	366(39.5)	667(31.6)	583(30.2)	566(28.1)	479(20.8)	518(17.8)	254(16.6)	65(17.2)	<0.001
	(N=790)	(N=1875)	(N=1891)	(N=1970)	(N=1856)	(N=1772)	(N=725)	(N=196)	
Female	298(37.7)	669(35.7)	774(40.9)	774(39.3)	660(35.6)	573(32.3)	200(27.6)	59(30.1)	<0.001
Coefficient	0.259	3.65	22.9	28.0	63.0	79.1	23.5	8.01	
P value	0.611	0.056	<0.001	<0.001	<0.001	<0.001	<0.001	0.004	
