# Supplementary material for: Proportionate clinical burden of respiratory diseases in Indian outdoor services and its relationship with seasonal transitions and risk factors: The results of SWORD survey
Source: PLoS One. 2022 Aug 18;17(8):e0268216. doi: 10.1371/journal.pone.0268216 (PMC9387816; doi:10.1371/journal.pone.0268216)
Supplement: S2 File — (PDF) [file pone.0268216.s002.pdf]

SWORD India : The point prevalence survey

(<u>S</u>easonal <u>W</u>aves <u>o</u>f <u>R</u>espiratory <u>D</u>isorders in <u>India</u>)

A joint venture of Indian Chest Society & Asthma Bhawan

Center Code: ____________

Name of consultant

(Capital letters) ____

Qualification (tick √ underlined text)

<u>DTCD</u> <u>MD-Chest</u> <u>MD-Medicine</u> <u>DM</u> <u>DNB</u>/Other:

City:

PIN Code:

Type of practice (tick √ underlined text)

State:

<u>Med.-College</u> <u>District-hospital</u> <u>Private-Clinic</u> /Other:

Date / / 201

Average daily temperature of city (on your cell phone) _________ ˚C

Tick √ the appropriate box below if yes to a variable (factor) listed on the left for patient 1 – 20

NOTE: Keep method and place of recruitment same in all SWORD phases. I took (a) all patients, (b) alternate patients

[First column 'Ex.= example' shows how to fill the boxes, watermarks will help you locate the boxes]

DAY 1 PART-A Preclinical Assessment

	**Demographic Factors**	**Ex.**	**1**	**2**	**3**	**4**	**5**	**6**	**7**	**8**	**9**	**10**	**11**	**12**	**13**	**14**	**15**	**16**	**17**	**18**	**19**	**20**
1	Age in years	44																				
2	Duration of disease – D=Days, W= Wks, M= Mths, Y=Yrs	5Y																				
3	Income- Low (below poverty line)	√	1	2	3	4	5	6	7	8	9	10	11	12	13	14	15	16	17	18	19	20
4	Female		1	2	3	4	5	6	7	8	9	10	11	12	13	14	15	16	17	18	19	20
5	Male	√	1	2	3	4	5	6	7	8	9	10	11	12	13	14	15	16	17	18	19	20
6	Pregnancy		1	2	3	4	5	6	7	8	9	10	11	12	13	14	15	16	17	18	19	20
	**Vaccination**	**Ex.**	**1**	**2**	**3**	**4**	**5**	**6**	**7**	**8**	**9**	**10**	**11**	**12**	**13**	**14**	**15**	**16**	**17**	**18**	**19**	**20**
7	Flu vaccine - last one year		1	2	3	4	5	6	7	8	9	10	11	12	13	14	15	16	17	18	19	20
8	Pneumococcal vaccine any time		1	2	3	4	5	6	7	8	9	10	11	12	13	14	15	16	17	18	19	20
	**Drug History**	**Ex.**	**1**	**2**	**3**	**4**	**5**	**6**	**7**	**8**	**9**	**10**	**11**	**12**	**13**	**14**	**15**	**16**	**17**	**18**	**19**	**20**
9	Anti-tuberculosis drugs – Took complete ATT		1	2	3	4	5	6	7	8	9	10	11	12	13	14	15	16	17	18	19	20
10	Anti-tuberculosis drugs – Took incomplete ATT	√	1	2	3	4	5	6	7	8	9	10	11	12	13	14	15	16	17	18	19	20
11	Taking anti-tuberculosis drugs		1	2	3	4	5	6	7	8	9	10	11	12	13	14	15	16	17	18	19	20
	**Risk Factors**	**Ex.**	**1**	**2**	**3**	**4**	**5**	**6**	**7**	**8**	**9**	**10**	**11**	**12**	**13**	**14**	**15**	**16**	**17**	**18**	**19**	**20**
12	Biomass fuel exposure – Cooking with wood /dung		1	2	3	4	5	6	7	8	9	10	11	12	13	14	15	16	17	18	19	20
13	Birds at home		1	2	3	4	5	6	7	8	9	10	11	12	13	14	15	16	17	18	19	20
14	Pets at home		1	2	3	4	5	6	7	8	9	10	11	12	13	14	15	16	17	18	19	20
15	Rain wetting (got wet in rain during last 1week)		1	2	3	4	5	6	7	8	9	10	11	12	13	14	15	16	17	18	19	20
16	Smoking – Ex-smoker Smokefree for at least 1 month		1	2	3	4	5	6	7	8	9	10	11	12	13	14	15	16	17	18	19	20
17	Smoker – E.g.,Bidi/cigarette/chillum/hookah	√	1	2	3	4	5	6	7	8	9	10	11	12	13	14	15	16	17	18	19	20
18	Travel – Recent Within 1 month		1	2	3	4	5	6	7	8	9	10	11	12	13	14	15	16	17	18	19	20
19	Visible mold at home /work place		1	2	3	4	5	6	7	8	9	10	11	12	13	14	15	16	17	18	19	20
20	Work in mine – (Stone/coal/gold)		1	2	3	4	5	6	7	8	9	10	11	12	13	14	15	16	17	18	19	20
	**Present Visit to Consultant**	**Ex.**	**1**	**2**	**3**	**4**	**5**	**6**	**7**	**8**	**9**	**10**	**11**	**12**	**13**	**14**	**15**	**16**	**17**	**18**	**19**	**20**
21	New visit	√	1	2	3	4	5	6	7	8	9	10	11	12	13	14	15	16	17	18	19	20
22	Revisit (follow-up)		1	2	3	4	5	6	7	8	9	10	11	12	13	14	15	16	17	18	19	20
23	Emergency visit		1	2	3	4	5	6	7	8	9	10	11	12	13	14	15	16	17	18	19	20
	**PIN (ZIP) Code**	**Ex.**	**1**	**2**	**3**	**4**	**5**	**6**	**7**	**8**	**9**	**10**	**11**	**12**	**13**	**14**	**15**	**16**	**17**	**18**	**19**	**20**
24	**Must enter PIN code** (If unable to enter PIN Write Village–District e.g., Chhiyal–Kullu **OR** Write Locality–City e.g., Shastri Nagar–Jaipur)	302016																				

DAY 1		PART-B Clinical Assessment														Center Code: __________					
	**Co morbid Conditions**	**Ex.**	**1**	**2**	**3**	**4**	**5**	**6**	**7**	**8**	**9**	**10**	**11**	**12**	**13**	**14**	**15**	**16**	**17**	**18**	**19**	**20**
25	Allergic Rhinitis		1	2	3	4	5	6	7	8	9	10	11	12	13	14	15	16	17	18	19	20
26	Anemia		1	2	3	4	5	6	7	8	9	10	11	12	13	14	15	16	17	18	19	20
27	Arthritis		1	2	3	4	5	6	7	8	9	10	11	12	13	14	15	16	17	18	19	20
28	Diabetes mellitus		1	2	3	4	5	6	7	8	9	10	11	12	13	14	15	16	17	18	19	20
29	Eczema		1	2	3	4	5	6	7	8	9	10	11	12	13	14	15	16	17	18	19	20
30	GERD (Gastroesophageal reflux)	√	1	2	3	4	5	6	7	8	9	10	11	12	13	14	15	16	17	18	19	20
31	Heart disease (coronary)		1	2	3	4	5	6	7	8	9	10	11	12	13	14	15	16	17	18	19	20
32	Hypertension		1	2	3	4	5	6	7	8	9	10	11	12	13	14	15	16	17	18	19	20
33	Urticaria		1	2	3	4	5	6	7	8	9	10	11	12	13	14	15	16	17	18	19	20
	**Presenting Symptoms**	**Ex.**	*1*	**2**	**3**	**4**	**5**	**6**	**7**	**8**	**9**	**10**	**11**	**12**	**13**	**14**	**15**	**16**	**17**	**18**	**19**	**20**
34	Breathlessness	√	1	2	3	4	5	6	7	8	9	10	11	12	13	14	15	16	17	18	19	20
35	Chest pain		1	2	3	4	5	6	7	8	9	10	11	12	13	14	15	16	17	18	19	20
36	Chest tightness		1	2	3	4	5	6	7	8	9	10	11	12	13	14	15	16	17	18	19	20
37	Cough- Productive	√	1	2	3	4	5	6	7	8	9	10	11	12	13	14	15	16	17	18	19	20
38	Cough- Dry		1	2	3	4	5	6	7	8	9	10	11	12	13	14	15	16	17	18	19	20
39	Fever	√	1	2	3	4	5	6	7	8	9	10	11	12	13	14	15	16	17	18	19	20
40	Hemoptysis		1	2	3	4	5	6	7	8	9	10	11	12	13	14	15	16	17	18	19	20
41	Pain in throat		1	2	3	4	5	6	7	8	9	10	11	12	13	14	15	16	17	18	19	20
42	Wheeze		1	2	3	4	5	6	7	8	9	10	11	12	13	14	15	16	17	18	19	20
	**Present Diagnosis**	**Ex.**	**1**	**2**	**3**	**4**	**5**	**6**	**7**	**8**	**9**	**10**	**11**	**12**	**13**	**14**	**15**	**16**	**17**	**18**	**19**	**20**
43	Asthma		1	2	3	4	5	6	7	8	9	10	11	12	13	14	15	16	17	18	19	20
44	Asthma- COPD overlap syndrome		1	2	3	4	5	6	7	8	9	10	11	12	13	14	15	16	17	18	19	20
45	Bronchiectasis- Post-tubercular		1	2	3	4	5	6	7	8	9	10	11	12	13	14	15	16	17	18	19	20
46	Bronchiectasis- ABPA		1	2	3	4	5	6	7	8	9	10	11	12	13	14	15	16	17	18	19	20
47	COPD		1	2	3	4	5	6	7	8	9	10	11	12	13	14	15	16	17	18	19	20
48	Hypersensitivity pneumonitis		1	2	3	4	5	6	7	8	9	10	11	12	13	14	15	16	17	18	19	20
49	Hyperventilation Syndrome		1	2	3	4	5	6	7	8	9	10	11	12	13	14	15	16	17	18	19	20
50	ILD– IPF		1	2	3	4	5	6	7	8	9	10	11	12	13	14	15	16	17	18	19	20
51	ILD– Other pattern		1	2	3	4	5	6	7	8	9	10	11	12	13	14	15	16	17	18	19	20
52	ILD– Collagen tissue disease		1	2	3	4	5	6	7	8	9	10	11	12	13	14	15	16	17	18	19	20
53	Lung cancer– NSCLC		1	2	3	4	5	6	7	8	9	10	11	12	13	14	15	16	17	18	19	20
54	Lung cancer– SCLC		1	2	3	4	5	6	7	8	9	10	11	12	13	14	15	16	17	18	19	20
55	Lung cancer– other		1	2	3	4	5	6	7	8	9	10	11	12	13	14	15	16	17	18	19	20
56	Pleural disease– Tubercular		1	2	3	4	5	6	7	8	9	10	11	12	13	14	15	16	17	18	19	20
57	Pleural disease– Other		1	2	3	4	5	6	7	8	9	10	11	12	13	14	15	16	17	18	19	20
58	Pneumoconiosis– Silicosis	√	1	2	3	4	5	6	7	8	9	10	11	12	13	14	15	16	17	18	19	20

		Ex.	1	2	3	4	5	6	7	8	9	10	11	12	13	14	15	16	17	18	19	20
59	Pneumoconiosis– Asbestosis		1	2	3	4	5	6	7	8	9	10	11	12	13	14	15	16	17	18	19	20
60	Pneumoconiosis– Coal worker pneumoconiosis		1	2	3	4	5	6	7	8	9	10	11	12	13	14	15	16	17	18	19	20
61	Pneumonia– Bacterial		1	2	3	4	5	6	7	8	9	10	11	12	13	14	15	16	17	18	19	20
62	Pneumonia– Viral		1	2	3	4	5	6	7	8	9	10	11	12	13	14	15	16	17	18	19	20
63	Pneumonia– Other		1	2	3	4	5	6	7	8	9	10	11	12	13	14	15	16	17	18	19	20
64	Post-tuberculosis COPD		1	2	3	4	5	6	7	8	9	10	11	12	13	14	15	16	17	18	19	20
65	Pulmonary aspergilloma		1	2	3	4	5	6	7	8	9	10	11	12	13	14	15	16	17	18	19	20
66	Pulmonary embolism		1	2	3	4	5	6	7	8	9	10	11	12	13	14	15	16	17	18	19	20
67	Pulmonary eosinophilia		1	2	3	4	5	6	7	8	9	10	11	12	13	14	15	16	17	18	19	20
68	Sarcoidosis		1	2	3	4	5	6	7	8	9	10	11	12	13	14	15	16	17	18	19	20
69	Sleep apnea		1	2	3	4	5	6	7	8	9	10	11	12	13	14	15	16	17	18	19	20
70	Tuberculosis- Newly diagnosed		1	2	3	4	5	6	7	8	9	10	11	12	13	14	15	16	17	18	19	20
71	Tuberculosis- Retreatment	√	1	2	3	4	5	6	7	8	9	10	11	12	13	14	15	16	17	18	19	20
72	Tuberculosis- MDR=M, XDR=X		1	2	3	4	5	6	7	8	9	10	11	12	13	14	15	16	17	18	19	20
73	URTI (Upper respiratory tract infection)		1	2	3	4	5	6	7	8	9	10	11	12	13	14	15	16	17	18	19	20
74	Other diagnosis – not listed above	Kyphoscoloiosis																				
	**Tests- (done/advised= √)**	**Ex.**	**1**	**2**	**3**	**4**	**5**	**6**	**7**	**8**	**9**	**10**	**11**	**12**	**13**	**14**	**15**	**16**	**17**	**18**	**19**	**20**
75	AFB smear	√	1	2	3	4	5	6	7	8	9	10	11	12	13	14	15	16	17	18	19	20
76	Arterial blood gas analysis		1	2	3	4	5	6	7	8	9	10	11	12	13	14	15	16	17	18	19	20
77	Bronchoscopy		1	2	3	4	5	6	7	8	9	10	11	12	13	14	15	16	17	18	19	20
78	Complete blood count with differential count		1	2	3	4	5	6	7	8	9	10	11	12	13	14	15	16	17	18	19	20
79	CBNAAT		1	2	3	4	5	6	7	8	9	10	11	12	13	14	15	16	17	18	19	20
80	Chest X–ray	√	1	2	3	4	5	6	7	8	9	10	11	12	13	14	15	16	17	18	19	20
81	CT of chest	√	1	2	3	4	5	6	7	8	9	10	11	12	13	14	15	16	17	18	19	20
82	DLco		1	2	3	4	5	6	7	8	9	10	11	12	13	14	15	16	17	18	19	20
83	FeNO (Exhaled breath nitric oxide)		1	2	3	4	5	6	7	8	9	10	11	12	13	14	15	16	17	18	19	20
84	Six-minute walk test		1	2	3	4	5	6	7	8	9	10	11	12	13	14	15	16	17	18	19	20
85	Sleep study		1	2	3	4	5	6	7	8	9	10	11	12	13	14	15	16	17	18	19	20
86	Spirometry		1	2	3	4	5	6	7	8	9	10	11	12	13	14	15	16	17	18	19	20
87	Other tests – not listed above	LFT, RFT																				

Center Code

Total no. of OPD patients (all consultants today) =

SWORD India (Seasonal Waves of Respiratory Disorders in India)

Tick √ the appropriate box below if yes to a variable (factor) listed on the left for patient 21 – 40.

[First column 'Ex.= example' shows how to fill the boxes]

	**DAY 1**	**PART-A**	**Preclinical Assessment**											**Center Code: __________**								
	**Demographic Factors**	**Ex.**	**21**	**22**	**23**	**24**	**25**	**26**	**27**	**28**	**29**	**30**	**31**	**32**	**33**	**34**	**35**	**36**	**37**	**38**	**39**	**40**
1	Age in years	**44**																				
2	Duration of disease – D=Days, W= Wks, M= Mths, Y=Yrs	**5Y**																				
3	Income- Low (below poverty line)	**√**	*21*	*22*	*23*	*24*	*25*	*26*	*27*	*28*	*29*	*30*	*31*	*32*	*33*	*34*	*35*	*36*	*37*	*38*	*39*	*40*
4	Female		*21*	*22*	*23*	*24*	*25*	*26*	*27*	*28*	*29*	*30*	*31*	*32*	*33*	*34*	*35*	*36*	*37*	*38*	*39*	*40*
5	Male	**√**	*21*	*22*	*23*	*24*	*25*	*26*	*27*	*28*	*29*	*30*	*31*	*32*	*33*	*34*	*35*	*36*	*37*	*38*	*39*	*40*
6	Pregnancy		*21*	*22*	*23*	*24*	*25*	*26*	*27*	*28*	*29*	*30*	*31*	*32*	*33*	*34*	*35*	*36*	*37*	*38*	*39*	*40*
	**Vaccination**	**Ex.**	**21**	**22**	**23**	**24**	**25**	**26**	**27**	**28**	**29**	**30**	**31**	**32**	**33**	**34**	**35**	**36**	**37**	**38**	**39**	**40**
7	Flu vaccine - last one year		*21*	*22*	*23*	*24*	*25*	*26*	*27*	*28*	*29*	*30*	*31*	*32*	*33*	*34*	*35*	*36*	*37*	*38*	*39*	*40*
8	Pneumococcal vaccine any time		*21*	*22*	*23*	*24*	*25*	*26*	*27*	*28*	*29*	*30*	*31*	*32*	*33*	*34*	*35*	*36*	*37*	*38*	*39*	*40*
	**Drug History**	**Ex.**	**21**	**22**	**23**	**24**	**25**	**26**	**27**	**28**	**29**	**30**	**31**	**32**	**33**	**34**	**35**	**36**	**37**	**38**	**39**	**40**
9	Anti-tuberculosis drugs – Took complete ATT		*21*	*22*	*23*	*24*	*25*	*26*	*27*	*28*	*29*	*30*	*31*	*32*	*33*	*34*	*35*	*36*	*37*	*38*	*39*	*40*
10	Anti-tuberculosis drugs – Took incomplete ATT	**√**	*21*	*22*	*23*	*24*	*25*	*26*	*27*	*28*	*29*	*30*	*31*	*32*	*33*	*34*	*35*	*36*	*37*	*38*	*39*	*40*
11	Taking anti-tuberculosis drugs		*21*	*22*	*23*	*24*	*25*	*26*	*27*	*28*	*29*	*30*	*31*	*32*	*33*	*34*	*35*	*36*	*37*	*38*	*39*	*40*
	**Risk Factors**	**Ex.**	**21**	**22**	**23**	**24**	**25**	**26**	**27**	**28**	**29**	**30**	**31**	**32**	**33**	**34**	**35**	**36**	**37**	**38**	**39**	**40**
12	Biomass fuel exposure – Cooking with wood /dung		*21*	*22*	*23*	*24*	*25*	*26*	*27*	*28*	*29*	*30*	*31*	*32*	*33*	*34*	*35*	*36*	*37*	*38*	*39*	*40*
13	Birds at home		*21*	*22*	*23*	*24*	*25*	*26*	*27*	*28*	*29*	*30*	*31*	*32*	*33*	*34*	*35*	*36*	*37*	*38*	*39*	*40*
14	Pets at home		*21*	*22*	*23*	*24*	*25*	*26*	*27*	*28*	*29*	*30*	*31*	*32*	*33*	*34*	*35*	*36*	*37*	*38*	*39*	*40*
15	Rain wetting (got wet in rain during last 1week)		*21*	*22*	*23*	*24*	*25*	*26*	*27*	*28*	*29*	*30*	*31*	*32*	*33*	*34*	*35*	*36*	*37*	*38*	*39*	*40*
16	Smoking – Ex-smoker Smokefree for at least 1 month		*21*	*22*	*23*	*24*	*25*	*26*	*27*	*28*	*29*	*30*	*31*	*32*	*33*	*34*	*35*	*36*	*37*	*38*	*39*	*40*
17	Smoker – E.g.,Bidi/cigarette/chillum/hookah	**√**	*21*	*22*	*23*	*24*	*25*	*26*	*27*	*28*	*29*	*30*	*31*	*32*	*33*	*34*	*35*	*36*	*37*	*38*	*39*	*40*
18	Travel-Recent Within 1 month		*21*	*22*	*23*	*24*	*25*	*26*	*27*	*28*	*29*	*30*	*31*	*32*	*33*	*34*	*35*	*36*	*37*	*38*	*39*	*40*
19	Visible mold at home /work place		*21*	*22*	*23*	*24*	*25*	*26*	*27*	*28*	*29*	*30*	*31*	*32*	*33*	*34*	*35*	*36*	*37*	*38*	*39*	*40*
20	Work in mine – (Stone/coal/gold)		*21*	*22*	*23*	*24*	*25*	*26*	*27*	*28*	*29*	*30*	*31*	*32*	*33*	*34*	*35*	*36*	*37*	*38*	*39*	*40*
	**Present Visit to Consultant**	**Ex.**	**21**	**22**	**23**	**24**	**25**	**26**	**27**	**28**	**29**	**30**	**31**	**32**	**33**	**34**	**35**	**36**	**37**	**38**	**39**	**40**
21	New visit	**√**	*21*	*22*	*23*	*24*	*25*	*26*	*27*	*28*	*29*	*30*	*31*	*32*	*33*	*34*	*35*	*36*	*37*	*38*	*39*	*40*
22	Revisit (follow-up)		*21*	*22*	*23*	*24*	*25*	*26*	*27*	*28*	*29*	*30*	*31*	*32*	*33*	*34*	*35*	*36*	*37*	*38*	*39*	*40*
23	Emergency visit		*21*	*22*	*23*	*24*	*25*	*26*	*27*	*28*	*29*	*30*	*31*	*32*	*33*	*34*	*35*	*36*	*37*	*38*	*39*	*40*
	**PIN (ZIP) Code**	**Ex.**	**21**	**22**	**23**	**24**	**25**	**26**	**27**	**28**	**29**	**30**	**31**	**32**	**33**	**34**	**35**	**36**	**37**	**38**	**39**	**40**
24	**Must enter PIN code** (If unable to enter PIN Write Village–District e.g., Chhiyal–Kullu **OR** Write Locality–City e.g., Shastri Nagar–Jaipur)	**302016**																				

DAY 1 **PART-B** **Clinical Assessment** **Center Code: __________**

	Co morbid Conditions	Ex.	21	22	23	24	25	26	27	28	29	30	31	32	33	34	35	36	37	38	39	40
25	Allergic Rhinitis		21	22	23	24	25	26	27	28	29	30	31	32	33	34	35	36	37	38	39	40
26	Anemia		21	22	23	24	25	26	27	28	29	30	31	32	33	34	35	36	37	38	39	40
27	Arthritis		21	22	23	24	25	26	27	28	29	30	31	32	33	34	35	36	37	38	39	40
28	Diabetes mellitus		21	22	23	24	25	26	27	28	29	30	31	32	33	34	35	36	37	38	39	40
29	Eczema		21	22	23	24	25	26	27	28	29	30	31	32	33	34	35	36	37	38	39	40
30	GERD (Gastroesophageal reflux)	√	21	22	23	24	25	26	27	28	29	30	31	32	33	34	35	36	37	38	39	40
31	Heart disease (coronary)		21	22	23	24	25	26	27	28	29	30	31	32	33	34	35	36	37	38	39	40
32	Hypertension		21	22	23	24	25	26	27	28	29	30	31	32	33	34	35	36	37	38	39	40
33	Urticaria		21	22	23	24	25	26	27	28	29	30	31	32	33	34	35	36	37	38	39	40
	**Presenting Symptoms**	**Ex.**	**21**	**22**	**23**	**24**	**25**	**26**	**27**	**28**	**29**	**30**	**31**	**32**	**33**	**34**	**35**	**36**	**37**	**38**	**39**	**40**
34	Breathlessness	√	21	22	23	24	25	26	27	28	29	30	31	32	33	34	35	36	37	38	39	40
35	Chest pain		21	22	23	24	25	26	27	28	29	30	31	32	33	34	35	36	37	38	39	40
36	Chest tightness		21	22	23	24	25	26	27	28	29	30	31	32	33	34	35	36	37	38	39	40
37	Cough- Productive	√	21	22	23	24	25	26	27	28	29	30	31	32	33	34	35	36	37	38	39	40
38	Cough- Dry		21	22	23	24	25	26	27	28	29	30	31	32	33	34	35	36	37	38	39	40
39	Fever	√	21	22	23	24	25	26	27	28	29	30	31	32	33	34	35	36	37	38	39	40
40	Hemoptysis		21	22	23	24	25	26	27	28	29	30	31	32	33	34	35	36	37	38	39	40
41	Pain in throat		21	22	23	24	25	26	27	28	29	30	31	32	33	34	35	36	37	38	39	40
42	Wheeze		21	22	23	24	25	26	27	28	29	30	31	32	33	34	35	36	37	38	39	40
	**Present Diagnosis**	**Ex.**	**21**	**22**	**23**	**24**	**25**	**26**	**27**	**28**	**29**	**30**	**31**	**32**	**33**	**34**	**35**	**36**	**37**	**38**	**39**	**40**
43	Asthma		21	22	23	24	25	26	27	28	29	30	31	32	33	34	35	36	37	38	39	40
44	Asthma- COPD overlap syndrome		21	22	23	24	25	26	27	28	29	30	31	32	33	34	35	36	37	38	39	40
45	Bronchiectasis- Post-tubercular		21	22	23	24	25	26	27	28	29	30	31	32	33	34	35	36	37	38	39	40
46	Bronchiectasis- ABPA		21	22	23	24	25	26	27	28	29	30	31	32	33	34	35	36	37	38	39	40
47	COPD		21	22	23	24	25	26	27	28	29	30	31	32	33	34	35	36	37	38	39	40
48	Hypersensitivity pneumonitis		21	22	23	24	25	26	27	28	29	30	31	32	33	34	35	36	37	38	39	40
49	Hyperventilation Syndrome		21	22	23	24	25	26	27	28	29	30	31	32	33	34	35	36	37	38	39	40
50	ILD– IPF		21	22	23	24	25	26	27	28	29	30	31	32	33	34	35	36	37	38	39	40
51	ILD– Other pattern		21	22	23	24	25	26	27	28	29	30	31	32	33	34	35	36	37	38	39	40
52	ILD– Collagen tissue disease		21	22	23	24	25	26	27	28	29	30	31	32	33	34	35	36	37	38	39	40
53	Lung cancer– NSCLC		21	22	23	24	25	26	27	28	29	30	31	32	33	34	35	36	37	38	39	40
54	Lung cancer– SCLC		21	22	23	24	25	26	27	28	29	30	31	32	33	34	35	36	37	38	39	40
55	Lung cancer– other		21	22	23	24	25	26	27	28	29	30	31	32	33	34	35	36	37	38	39	40
56	Pleural disease– Tubercular		21	22	23	24	25	26	27	28	29	30	31	32	33	34	35	36	37	38	39	40
57	Pleural disease– Other		21	22	23	24	25	26	27	28	29	30	31	32	33	34	35	36	37	38	39	40
58	Pneumoconiosis– Silicosis	√	21	22	23	24	25	26	27	28	29	30	31	32	33	34	35	36	37	38	39	40

Present diagnosis contd..

59	Pneumoconiosis– Asbestosis		21	22	23	24	25	26	27	28	29	30	31	32	33	34	35	36	37	38	39	40
60	Pneumoconiosis– Coal worker pneumoconiosis		21	22	23	24	25	26	27	28	29	30	31	32	33	34	35	36	37	38	39	40
61	Pneumonia– Bacterial		21	22	23	24	25	26	27	28	29	30	31	32	33	34	35	36	37	38	39	40
62	Pneumonia– Viral		21	22	23	24	25	26	27	28	29	30	31	32	33	34	35	36	37	38	39	40
63	Pneumonia– Other		21	22	23	24	25	26	27	28	29	30	31	32	33	34	35	36	37	38	39	40
64	Post-tuberculosis COPD		21	22	23	24	25	26	27	28	29	30	31	32	33	34	35	36	37	38	39	40
65	Pulmonary aspergilloma		21	22	23	24	25	26	27	28	29	30	31	32	33	34	35	36	37	38	39	40
66	Pulmonary embolism		21	22	23	24	25	26	27	28	29	30	31	32	33	34	35	36	37	38	39	40
67	Pulmonary eosinophilia		21	22	23	24	25	26	27	28	29	30	31	32	33	34	35	36	37	38	39	40
68	Sarcoidosis		21	22	23	24	25	26	27	28	29	30	31	32	33	34	35	36	37	38	39	40
69	Sleep apnea		21	22	23	24	25	26	27	28	29	30	31	32	33	34	35	36	37	38	39	40
70	Tuberculosis- Newly diagnosed		21	22	23	24	25	26	27	28	29	30	31	32	33	34	35	36	37	38	39	40
71	Tuberculosis- Retreatment	√	21	22	23	24	25	26	27	28	29	30	31	32	33	34	35	36	37	38	39	40
72	Tuberculosis- MDR=M, XDR=X		21	22	23	24	25	26	27	28	29	30	31	32	33	34	35	36	37	38	39	40
73	URTI (Upper respiratory tract infection)		21	22	23	24	25	26	27	28	29	30	31	32	33	34	35	36	37	38	39	40
74	Other diagnosis – not listed above	Kyphoscoloiosis																				
	**Tests- (done/advised= √)**	**Ex.**	**21**	**22**	**23**	**24**	**25**	**26**	**27**	**28**	**29**	**30**	**31**	**32**	**33**	**34**	**35**	**36**	**37**	**38**	**39**	**40**
75	AFB smear	√	21	22	23	24	25	26	27	28	29	30	31	32	33	34	35	36	37	38	39	40
76	Arterial blood gas analysis		21	22	23	24	25	26	27	28	29	30	31	32	33	34	35	36	37	38	39	40
77	Bronchoscopy		21	22	23	24	25	26	27	28	29	30	31	32	33	34	35	36	37	38	39	40
78	Complete blood count with differential count		21	22	23	24	25	26	27	28	29	30	31	32	33	34	35	36	37	38	39	40
79	CBNAAT		21	22	23	24	25	26	27	28	29	30	31	32	33	34	35	36	37	38	39	40
80	Chest X–ray	√	21	22	23	24	25	26	27	28	29	30	31	32	33	34	35	36	37	38	39	40
81	CT of chest	√	21	22	23	24	25	26	27	28	29	30	31	32	33	34	35	36	37	38	39	40
82	DLco		21	22	23	24	25	26	27	28	29	30	31	32	33	34	35	36	37	38	39	40
83	FeNO (Exhaled breath nitric oxide)		21	22	23	24	25	26	27	28	29	30	31	32	33	34	35	36	37	38	39	40
84	Six-minute walk test		21	22	23	24	25	26	27	28	29	30	31	32	33	34	35	36	37	38	39	40
85	Sleep study		21	22	23	24	25	26	27	28	29	30	31	32	33	34	35	36	37	38	39	40
86	Spirometry		21	22	23	24	25	26	27	28	29	30	31	32	33	34	35	36	37	38	39	40
87	Other tests – not listed above	LFT, RFT																				

SWORD India (Seasonal Waves of Respiratory Disorders in India)

Tick √ the appropriate box below if yes to a variable (factor) listed on the left for patient 41 – 60.

[First column 'Ex.=example' shows how to fill the boxes]

DAY 1 PART-A Preclinical Assessment Center Code: __________

	Demographic Factors	Ex.	41	42	43	44	45	46	47	48	49	50	51	52	53	54	55	56	57	58	59	60
1	Age in years	44																				
2	Duration of disease – D=Days, W= Wks, M= Mths, Y=Yrs	5Y																				
3	Income- Low (below poverty line)	√	41	42	43	44	45	46	47	48	49	50	51	52	53	54	55	56	57	58	59	60
4	Female		41	42	43	44	45	46	47	48	49	50	51	52	53	54	55	56	57	58	59	60
5	Male	√	41	42	43	44	45	46	47	48	49	50	51	52	53	54	55	56	57	58	59	60
6	Pregnancy		41	42	43	44	45	46	47	48	49	50	51	52	53	54	55	56	57	58	59	60
	**Vaccination**	Ex.	41	42	43	44	45	46	47	48	49	50	51	52	53	54	55	56	57	58	59	60
7	Flu vaccine - last one year		41	42	43	44	45	46	47	48	49	50	51	52	53	54	55	56	57	58	59	60
8	Pneumococcal vaccine any time		41	42	43	44	45	46	47	48	49	50	51	52	53	54	55	56	57	58	59	60
	**Drug History**	Ex.	41	42	43	44	45	46	47	48	49	50	51	52	53	54	55	56	57	58	59	60
9	Anti-tuberculosis drugs – Took complete ATT		41	42	43	44	45	46	47	48	49	50	51	52	53	54	55	56	57	58	59	60
10	Anti-tuberculosis drugs – Took incomplete ATT	√	41	42	43	44	45	46	47	48	49	50	51	52	53	54	55	56	57	58	59	60
11	Taking anti-tuberculosis drugs		41	42	43	44	45	46	47	48	49	50	51	52	53	54	55	56	57	58	59	60
	**Risk Factors**	Ex.	41	42	43	44	45	46	47	48	49	50	51	52	53	54	55	56	57	58	59	60
12	Biomass fuel exposure – Cooking with wood /dung		41	42	43	44	45	46	47	48	49	50	51	52	53	54	55	56	57	58	59	60
13	Birds at home		41	42	43	44	45	46	47	48	49	50	51	52	53	54	55	56	57	58	59	60
14	Pets at home		41	42	43	44	45	46	47	48	49	50	51	52	53	54	55	56	57	58	59	60
15	Rain wetting (got wet in rain during last 1week)		41	42	43	44	45	46	47	48	49	50	51	52	53	54	55	56	57	58	59	60
16	Smoking – Ex-smoker Smokefree for at least 1 month		41	42	43	44	45	46	47	48	49	50	51	52	53	54	55	56	57	58	59	60
17	Smoker– E.g.,Bidi/cigarette/chillum/hookah	√	41	42	43	44	45	46	47	48	49	50	51	52	53	54	55	56	57	58	59	60
18	Travel-Recent Within 1 month		41	42	43	44	45	46	47	48	49	50	51	52	53	54	55	56	57	58	59	60
19	Visible mold at home /work place		41	42	43	44	45	46	47	48	49	50	51	52	53	54	55	56	57	58	59	60
20	Work in mine – (Stone/coal/gold)		41	42	43	44	45	46	47	48	49	50	51	52	53	54	55	56	57	58	59	60
	**Present Visit to Consultant**	Ex.	41	42	43	44	45	46	47	48	49	50	51	52	53	54	55	56	57	58	59	60
21	New visit	√	41	42	43	44	45	46	47	48	49	50	51	52	53	54	55	56	57	58	59	60
22	Revisit (follow-up)		41	42	43	44	45	46	47	48	49	50	51	52	53	54	55	56	57	58	59	60
23	Emergency visit		41	42	43	44	45	46	47	48	49	50	51	52	53	54	55	56	57	58	59	60
	**PIN (ZIP) Code**	Ex.	41	42	43	44	45	46	47	48	49	50	51	52	53	54	55	56	57	58	59	60
24	**Must enter PIN code** (If unable to enter PIN Write Village–District e.g., Chhiyal–Kullu **OR** Write Locality–City e.g., Shastri Nagar–Jaipur)	3 0 2 0 1 6																				

DAY 1	PART-B Clinical Assessment																					Center Code: __________
	**Co morbid Conditions**	**Ex.**	**41**	**42**	**43**	**44**	**45**	**46**	**47**	**48**	**49**	**50**	**51**	**52**	**53**	**54**	**55**	**56**	**57**	**58**	**59**	**60**
25	Allergic Rhinitis		41	42	43	44	45	46	47	48	49	50	51	52	53	54	55	56	57	58	59	60
26	Anemia		41	42	43	44	45	46	47	48	49	50	51	52	53	54	55	56	57	58	59	60
27	Arthritis		41	42	43	44	45	46	47	48	49	50	51	52	53	54	55	56	57	58	59	60
28	Diabetes mellitus		41	42	43	44	45	46	47	48	49	50	51	52	53	54	55	56	57	58	59	60
29	Eczema		41	42	43	44	45	46	47	48	49	50	51	52	53	54	55	56	57	58	59	60
30	GERD (Gastroesophageal reflux)	√	41	42	43	44	45	46	47	48	49	50	51	52	53	54	55	56	57	58	59	60
31	Heart disease (coronary)		41	42	43	44	45	46	47	48	49	50	51	52	53	54	55	56	57	58	59	60
32	Hypertension		41	42	43	44	45	46	47	48	49	50	51	52	53	54	55	56	57	58	59	60
33	Urticaria		41	42	43	44	45	46	47	48	49	50	51	52	53	54	55	56	57	58	59	60
	**Presenting Symptoms**	**Ex.**	**41**	**42**	**43**	**44**	**45**	**46**	**47**	**48**	**49**	**50**	**51**	**52**	**53**	**54**	**55**	**56**	**57**	**58**	**59**	**60**
34	Breathlessness	√	41	42	43	44	45	46	47	48	49	50	51	52	53	54	55	56	57	58	59	60
35	Chest pain		41	42	43	44	45	46	47	48	49	50	51	52	53	54	55	56	57	58	59	60
36	Chest tightness		41	42	43	44	45	46	47	48	49	50	51	52	53	54	55	56	57	58	59	60
37	Cough- Productive	√	41	42	43	44	45	46	47	48	49	50	51	52	53	54	55	56	57	58	59	60
38	Cough- Dry		41	42	43	44	45	46	47	48	49	50	51	52	53	54	55	56	57	58	59	60
39	Fever	√	41	42	43	44	45	46	47	48	49	50	51	52	53	54	55	56	57	58	59	60
40	Hemoptysis		41	42	43	44	45	46	47	48	49	50	51	52	53	54	55	56	57	58	59	60
41	Pain in throat		41	42	43	44	45	46	47	48	49	50	51	52	53	54	55	56	57	58	59	60
42	Wheeze		41	42	43	44	45	46	47	48	49	50	51	52	53	54	55	56	57	58	59	60
	**Present Diagnosis**	**Ex.**	**41**	**42**	**43**	**44**	**45**	**46**	**47**	**48**	**49**	**50**	**51**	**52**	**53**	**54**	**55**	**56**	**57**	**58**	**59**	**60**
43	Asthma		41	42	43	44	45	46	47	48	49	50	51	52	53	54	55	56	57	58	59	60
44	Asthma- COPD overlap syndrome		41	42	43	44	45	46	47	48	49	50	51	52	53	54	55	56	57	58	59	60
45	Bronchiectasis- Post-tubercular		41	42	43	44	45	46	47	48	49	50	51	52	53	54	55	56	57	58	59	60
46	Bronchiectasis- ABPA		41	42	43	44	45	46	47	48	49	50	51	52	53	54	55	56	57	58	59	60
47	COPD		41	42	43	44	45	46	47	48	49	50	51	52	53	54	55	56	57	58	59	60
48	Hypersensitivity pneumonitis		41	42	43	44	45	46	47	48	49	50	51	52	53	54	55	56	57	58	59	60
49	Hyperventilation Syndrome		41	42	43	44	45	46	47	48	49	50	51	52	53	54	55	56	57	58	59	60
50	ILD– IPF		41	42	43	44	45	46	47	48	49	50	51	52	53	54	55	56	57	58	59	60
51	ILD– Other pattern		41	42	43	44	45	46	47	48	49	50	51	52	53	54	55	56	57	58	59	60
52	ILD– Collagen tissue disease		41	42	43	44	45	46	47	48	49	50	51	52	53	54	55	56	57	58	59	60
53	Lung cancer– NSCLC		41	42	43	44	45	46	47	48	49	50	51	52	53	54	55	56	57	58	59	60
54	Lung cancer– SCLC		41	42	43	44	45	46	47	48	49	50	51	52	53	54	55	56	57	58	59	60
55	Lung cancer– other		41	42	43	44	45	46	47	48	49	50	51	52	53	54	55	56	57	58	59	60
56	Pleural disease– Tubercular		41	42	43	44	45	46	47	48	49	50	51	52	53	54	55	56	57	58	59	60
57	Pleural disease– Other		41	42	43	44	45	46	47	48	49	50	51	52	53	54	55	56	57	58	59	60
58	Pneumoconiosis– Silicosis	√	41	42	43	44	45	46	47	48	49	50	51	52	53	54	55	56	57	58	59	60

Present diagnosis contd..

59	Pneumoconiosis– Asbestosis		41	42	43	44	45	46	47	48	49	50	51	52	53	54	55	56	57	58	59	60
60	Pneumoconiosis– Coal worker pneumoconiosis		41	42	43	44	45	46	47	48	49	50	51	52	53	54	55	56	57	58	59	60
61	Pneumonia– Bacterial		41	42	43	44	45	46	47	48	49	50	51	52	53	54	55	56	57	58	59	60
62	Pneumonia– Viral		41	42	43	44	45	46	47	48	49	50	51	52	53	54	55	56	57	58	59	60
63	Pneumonia– Other		41	42	43	44	45	46	47	48	49	50	51	52	53	54	55	56	57	58	59	60
64	Post-tuberculosis COPD		41	42	43	44	45	46	47	48	49	50	51	52	53	54	55	56	57	58	59	60
65	Pulmonary aspergilloma		41	42	43	44	45	46	47	48	49	50	51	52	53	54	55	56	57	58	59	60
66	Pulmonary embolism		41	42	43	44	45	46	47	48	49	50	51	52	53	54	55	56	57	58	59	60
67	Pulmonary eosinophilia		41	42	43	44	45	46	47	48	49	50	51	52	53	54	55	56	57	58	59	60
68	Sarcoidosis		41	42	43	44	45	46	47	48	49	50	51	52	53	54	55	56	57	58	59	60
69	Sleep apnea		41	42	43	44	45	46	47	48	49	50	51	52	53	54	55	56	57	58	59	60
70	Tuberculosis- Newly diagnosed		41	42	43	44	45	46	47	48	49	50	51	52	53	54	55	56	57	58	59	60
71	Tuberculosis- Retreatment	√	41	42	43	44	45	46	47	48	49	50	51	52	53	54	55	56	57	58	59	60
72	Tuberculosis- MDR=M, XDR=X		41	42	43	44	45	46	47	48	49	50	51	52	53	54	55	56	57	58	59	60
73	URTI (Upper respiratory tract infection)		41	42	43	44	45	46	47	48	49	50	51	52	53	54	55	56	57	58	59	60
74	Other diagnosis – not listed above	Kyphoscoloiosis																				
	**Tests- (done/advised= √)**	**Ex.**	**41**	**42**	**43**	**44**	**45**	**46**	**47**	**48**	**49**	**50**	**51**	**52**	**53**	**54**	**55**	**56**	**57**	**58**	**59**	**60**
75	AFB smear	√	41	42	43	44	45	46	47	48	49	50	51	52	53	54	55	56	57	58	59	60
76	Arterial blood gas analysis		41	42	43	44	45	46	47	48	49	50	51	52	53	54	55	56	57	58	59	60
77	Bronchoscopy		41	42	43	44	45	46	47	48	49	50	51	52	53	54	55	56	57	58	59	60
78	Complete blood count with differential count		41	42	43	44	45	46	47	48	49	50	51	52	53	54	55	56	57	58	59	60
79	CBNAAT		41	42	43	44	45	46	47	48	49	50	51	52	53	54	55	56	57	58	59	60
80	Chest X–ray	√	41	42	43	44	45	46	47	48	49	50	51	52	53	54	55	56	57	58	59	60
81	CT of chest	√	41	42	43	44	45	46	47	48	49	50	51	52	53	54	55	56	57	58	59	60
82	DLco		41	42	43	44	45	46	47	48	49	50	51	52	53	54	55	56	57	58	59	60
83	FeNO (Exhaled breath nitric oxide)		41	42	43	44	45	46	47	48	49	50	51	52	53	54	55	56	57	58	59	60
84	Six-minute walk test		41	42	43	44	45	46	47	48	49	50	51	52	53	54	55	56	57	58	59	60
85	Sleep study		41	42	43	44	45	46	47	48	49	50	51	52	53	54	55	56	57	58	59	60
86	Spirometry		41	42	43	44	45	46	47	48	49	50	51	52	53	54	55	56	57	58	59	60
87	Other tests – not listed above	LFT, RFT																				

Center Code

Total no. of OPD patients (all consultants today) =
